# Design and Synthesis of Imidazole and Triazole Pyrazoles as *Mycobacterium Tuberculosis* CYP121A1 Inhibitors

**DOI:** 10.1002/open.201900227

**Published:** 2019-07-23

**Authors:** Safaa M. Kishk, Kirsty J. McLean, Sakshi Sood, Darren Smith, Jack W.D. Evans, Mohamed A. Helal, Mohamed S. Gomaa, Ismail Salama, Samia M. Mostafa, Luiz Pedro S. de Carvalho, Colin W. Levy, Andrew W. Munro, Claire Simons

**Affiliations:** ^1^ School of Pharmacy & Pharmaceutical Sciences Cardiff University King Edward VII Avenue Cardiff CF10 3NB U.K.; ^2^ Medicinal Chemistry Department, Faculty of Pharmacy Suez Canal University Ismailia Egypt; ^3^ Manchester Institute of Biotechnology, School of Chemistry University of Manchester 131 Princess Street Manchester M1 7DN U.K.; ^4^ Mycobacterial Metabolism and Antibiotic Research Laboratory The Francis Crick Institute 1 Midland Road London NW1 1AT U.K.; ^5^ Biomedical Sciences Program University of Science and Technology Zewail City of Science and Technology Giza 12588 Egypt; ^6^ Department of Chemistry College of Clinical Pharmacy Imam Abdulrahman Bin Faisal University Dammam Kingdom of Saudi Arabia

**Keywords:** *mycobacterium tuberculosis*, Imidazole derivatives, Binding affinity, molecular modelling, X-ray crystallography

## Abstract

The emergence of untreatable drug‐resistant strains of *Mycobacterium tuberculosis* is a major public health problem worldwide, and the identification of new efficient treatments is urgently needed. *Mycobacterium tuberculosis* cytochrome P450 CYP121A1 is a promising drug target for the treatment of tuberculosis owing to its essential role in mycobacterial growth. Using a rational approach, which includes molecular modelling studies, three series of azole pyrazole derivatives were designed through two synthetic pathways. The synthesized compounds were biologically evaluated for their inhibitory activity towards *M. tuberculosis* and their protein binding affinity (*K*_D_). Series 3 biarylpyrazole imidazole derivatives were the most effective with the isobutyl (**10 f**) and *tert*‐butyl (**10 g**) compounds displaying optimal activity (MIC 1.562 μg/mL, *K*_D_ 0.22 μM (**10 f**) and 4.81 μM (**10 g**)). The spectroscopic data showed that all the synthesised compounds produced a type II red shift of the heme Soret band indicating either direct binding to heme iron or (where less extensive Soret shifts are observed) putative indirect binding via an interstitial water molecule. Evaluation of biological and physicochemical properties identified the following as requirements for activity: LogP >4, H‐bond acceptors/H‐bond donors 4/0, number of rotatable bonds 5–6, molecular volume >340 Å^3^, topological polar surface area <40 Å^2^.

## 1 Introduction

Tuberculosis (TB) is the ninth leading cause of death worldwide and the leading cause from a single infectious agent, ranking above HIV/AIDS.^1^ In 2016, 6.3 million new cases of TB were reported; of these there were an estimated 1.3 million deaths among HIV‐negative people and an estimated 374,000 deaths among HIV‐positive people.^1^ Drug‐resistant TB is a continuing challenge, with an estimated 4.1 % of new cases and 19 % of previously treated cases presenting with multidrug resistant (MDR)/rifampicin resistant (RR) TB in 2016.^1^ China, India and the Russian Federation have the largest number of MDR/RR‐TB cases accounting for 47 % of the global total.^1^

Drugs currently used to treat TB target cell wall synthesis (*e. g*. isoniazid, ethambutol, cycloserine), protein synthesis (*e. g*. capreomycin, kanamycin), RNA synthesis (*e. g*. rifampicin), DNA gyrase (*e. g*. fluoroquinolones) and ATP synthase (*e. g*. bedaquiline and delamanid) in *Mycobacterium tuberculosis* (Mtb), the causative agent of TB.^2^ The first line TB drug regimen includes a combination of isoniazid, ethambutol, rifampicin and pyrazinamide over a 6–9 month period and, whilst effective, this therapy is associated with drug intolerance and toxicity.^3^ Treatment of MDR‐TB and extensively drug resistant (XDR) TB is more complex and also associated with increased side effects.^2^, ^4^ The emergence of MDR‐TB, XDR‐TB and totally drug resistant (TDR) Mtb strains has led to intensified research to identify new anti‐TB drugs over the past decade.

**Figure 1:**
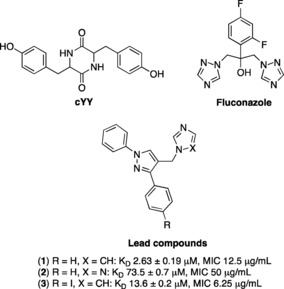
Lead compounds (**1**–**3**), the natural substrate cYY and CYP121A1 inhibitor fluconazole.

**Figure 2:**
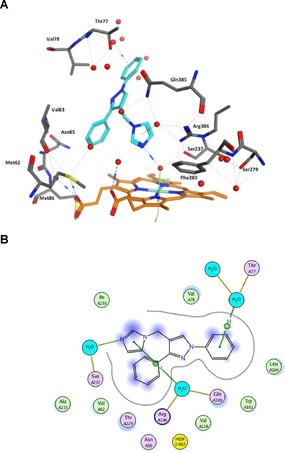
(A) 3D and (B) 2D images of the binding interactions of lead compound **1** in the CYP121A1 active site. Binding with the heme is through an interstitial water molecule with Ser237, Gln385, Arg386 and Thr77 as key binding amino acids.

Mtb encodes twenty cytochrome P450 enzymes (CYPs or P450s), three of these (CYP121A1, CYP125 A1 and CYP128 A1) were shown to be essential for mycobacterial growth or survival in the host.^5^ CYP121A1 is of considerable interest from both a biochemical and drug design perspective. CYP121A1 catalyses a unique C−C bond formation between carbon atoms in the *ortho*‐positions of two tyrosine groups in the cyclodipeptide dicyclotyrosine (cYY, Figure 1), the natural substrate, to form the metabolite mycocyclosin.^6^ The role of mycocylosin in Mtb remains unclear, although cyclodipeptides have important biological effects, such as inhibition of production of bacterial virulence factors and biofilm formation.^7^, ^8^ Drug design for CYP121A1 inhibitors has followed several approaches: (1) a fragment‐based approach has been very effective in developing compounds with low nM enzyme binding affinity, however this has not translated to antimycobacterial activity;^9^, ^10^ (2) a recent biophysical screening of a library of described P450 inhibitors has led to a promising lead compound with both good binding affinity and antimycobacterial activity;^11^ (3) our own approach has focused on developing a series of different compound scaffolds as mimics of cYY, resulting in both good binding affinity and promising antimycobacterial activity.^12^, ^13^ Our previous research identified derivatives of (1,3‐diphenyl)‐4‐((1,3‐imidazol‐1‐yl)methyl)‐1*H*‐pyrazole and 1‐((1,3‐diphenyl‐1*H*‐pyrazol‐4‐yl)methyl)‐1*H*‐1, 2, 4triazole as inhibitors of CYP121A1 (*e. g*. **1**, **2** and **3**, Figure 1),^13^ representing lead compounds for further development. Diphenyl pyrazole derivatives were found to interact with the active site of CYP121A1 in a manner similar to fluconazole and the natural substrate cYY (Figure 1), interacting indirectly with the heme via an interstitial water molecule (Figure 2).

**Figure 3:**
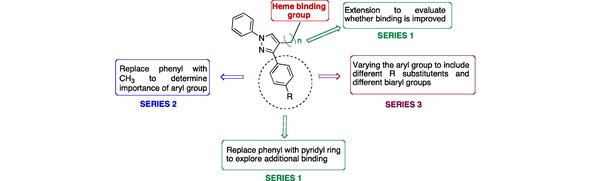
Modifications of the lead compounds, generating Series 1, 2 and 3

Introduction of a N atom in one of the phenyl rings, i. e. a pyridine ring, can potentially result in additional binding interactions, which may improve binding affinity. Likewise, extension of the chain between the pyrazole and the heme prosthetic group may allow the molecule to interact directly with the heme: this is the basis for Series 1 (Figure 3). The requirement for two phenyl rings was also explored in Series 2 by preparation of a small series of imidazole/triazole derivatives, with one of the phenyl rings replaced with a methyl group (Figure 3). Finally, our previous research showed a direct correlation between lipophilicity and MIC.^13^ Therefore in Series 3 one of the phenyl rings is further substituted with a range of alkyl substituted phenyl and biaryl groups to determine structure‐activity relationships (Figure 3).

## 2 Results and Discussion

### 2.1 Chemistry

**Scheme 1:**
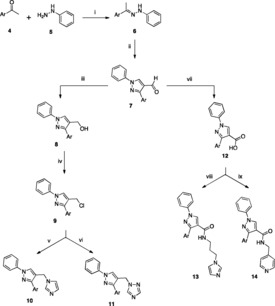
*Reagents and Conditions*: (i) AcOH, EtOH, 80 °C, 1 h (ii) POCl_3_, DMF, 60 °C, 4 h (iii) NaBH_4_, EtOH, r.t. 1 h (iv) SOCl_2_, CH_2_Cl_2_, r.t. overnight (v) imidazole, K_2_CO_3_, CH_3_CN, 45 °C, 1 h then 70 °C, overnight (vi) triazole, K_2_CO_3_, CH_3_CN, 45 °C, 1 h then 70 °C, overnight (vii) KMnO_4_, acetone/H_2_O, r.t. 1.5 h (viii) (a) CDI, DMF, r.t. 2 h (b) 1‐(3‐aminopropyl)imidazole, 70 °C, 48 h (ix) (a) CDI, DMF, r.t. 2 h (b) 4‐(aminomethyl)pyridine, 70 °C, 48 h. [**a**, Ar=4‐pyridyl; **b**, Ar=3‐pyridyl]

Series 1 pyridylpyrazole imidazole compounds (**10** and **11**) were obtained via a five‐step synthetic route (Scheme 1) beginning with the preparation of the imines (**6**)^14^ on reaction of the appropriate 3‐ or 4‐acetylpyridine reagent (**4**) with phenylhydrazine (**5**) under acidic conditions.^15^, ^16^ The yields obtained for the imines (**6**) were 98 and 97 % respectively for the 4‐pyridyl (**6 a**) and 3‐pyridyl (**6 b**) imines. The aldehydes (**7**) were prepared as previously described^14^ by a Vilsmeier‐Haack reaction of the imines (**6**).

Reduction of the aldehydes (**7**) with NaBH_4_ resulted in high yields of the corresponding alcohols (**8**). Thionyl chloride was the chlorinating reagent used to convert the alcohols to the chlorides (**9**), different equivalents of thionyl chloride were used to optimize the reaction conditions (1, 4 and 10 equivalents), and the maximum yield was obtained when 10 equivalents of the reagent were used. Reaction of the chlorides (**9**) with either the potassium salt of imidazole or triazole, prepared *in situ* by treatment of imidazole or triazole with potassium carbonate in acetonitrile at 45 °C for 1 h,^12^, ^13^ and overnight reflux at 70 °C gave the required final imidazole (**10 a** and **10 b**) and triazole (**11 a** and **11 b**) pyridyl‐pyrazole derivatives (Scheme 1) in yields of 67, 54, 48 and 55 % respectively.

To prepare the extended compounds, the aldehydes (**7**) were oxidized with potassium permanganate to give the carboxylic acids (**12**).^14^ The carboxylic acids were then coupled with either 1‐(3‐aminopropyl)imidazole or 4‐(aminomethyl)pyridine, after activation of the carboxylic acid with carbonyldiimidazole, to give the *N*‐(1*H*‐imidazolyl)propyl)‐1‐phenyl‐3‐(pyridyl)‐1*H*‐pyrazole‐4‐carboxamides (**13 a** and **13 b**) and 1‐phenyl‐3‐(pyridyl)‐*N*‐(pyridin‐4‐ylmethyl)‐1*H*‐pyrazole‐4‐carboxamides (**14 a** and **14 b**) (Scheme 1).

In series 2, one of the phenyl rings was replaced with a methyl group to determine whether the second phenyl ring was significant for enzyme binding and inhibitory activity.

**Scheme 2:**
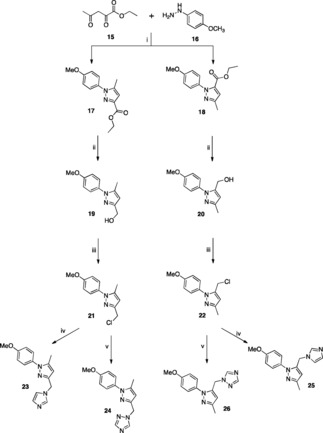
*Reagents and Conditions*: (i) EtOH, 80 °C, overnight (ii) LiAlH_4_, THF, r.t. overnight (iii) SOCl_2_, CH_2_Cl_2_, r.t. overnight (iv) imidazole, K_2_CO_3_, CH_3_CN, 45 °C, 1 h then 70 °C, overnight (v) triazole, K_2_CO_3_, CH_3_CN, 45 °C, 1 h then 70 °C, overnight.

For the remaining phenyl ring the 4‐methoxy phenyl group was chosen to more closely mimic the phenol ring of cYY (Figure 1). The 3/5‐((1*H*‐imidazol‐1‐yl)methyl)‐1‐(4‐methoxyphenyl)‐5/3‐methyl‐1*H*‐pyrazoles (**23** and **25**) and 1‐((1‐(4‐methoxyphenyl)‐5/3‐methyl‐1*H*‐pyrazol‐3/5‐yl)methyl)‐1*H*‐1,2,4‐triazoles (**24** and **26**) were prepared in four steps (Scheme 2). Formation of the pyrazole ethyl esters (**17** and **18**) was achieved by the reaction of ethyl‐2,4‐dioxovalerate (**15**) with 4‐methoxyphenyl hydrazine (**16**) by a Knorr pyrazole synthesis mechanism^17^ as previously described.^18^ The two regioisomers, ethyl 1‐(4‐methoxyphenyl)‐5‐methyl‐1*H*‐pyrazole‐3‐carboxylate (**17**) and ethyl 1‐(4‐methoxyphenyl)‐3‐methyl‐1*H*‐pyrazole‐5‐carboxylate (**18**) were obtained in a ratio of 1.3 : 1 and separated by column chromatography. The ethyl esters (**17** and **18**) were reduced to the alcohols **19** and **20** and then converted to the chlorides using thionyl chloride (**21** and **22**) followed by reaction with the potassium salt of imidazole or triazole, as previously described, to give the required final imidazole pyrazole derivatives (**23** and **25**) and triazole pyrazole derivatives (**24** and **26**) (Scheme 2).

The Series 3 biarylpyrazole imidazole (**10 c**‐**j**) compounds were obtained via the five‐step synthetic route described in Scheme 1 and were prepared to explore the effect of different aryl and biaryl groups on binding and their ‘fit’ within the active site, and also to evaluate the correlation between MIC and lipophilicity previously observed. Of note, although the yields obtained for the imines (**6 c–j**) varied between 84–99 %, they were generally found to be highly sensitive to light and moisture, especially those with low melting points, so were used immediately in the next step of the reaction scheme. Also, in the chlorination step of the indole derivative **8 j** a C‐2 chlorination of the indole ring occurred in addition to the conversion of the alcohol to chloride.

### 2.2 CYP121A1 Ligand Binding Affinity

**Figure 4:**
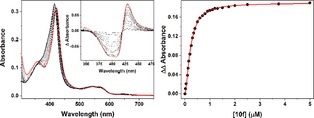
**Compound 10 f binding to CYP121A1 using UV‐Vis optical binding titration** The left‐hand panel shows spectra for compound **10 f** titrated with CYP121A1 (ligand‐free spectrum is a thick black line, spectra following progressive additions of compound **10 f** are thin solid lines, and the final near‐saturated protein spectrum is a thick red line). The inset shows overlapped difference spectra generated by subtracting the starting spectrum from each consecutive ligand‐bound spectrum. The right‐hand panel shows a plot of compound **10 f** induced‐absorbance change calculated as the difference between the peak and trough in the difference spectra in the left‐hand panel, using the same wavelength pair (429 and 392 nm, respectively). Data was fitted using the Hill equation giving a *K*_D_ value of 0.22±0.01 μM for **10 f**.

The CYP121A1 binding affinity (*K*_D_) of the various compounds was determined by UV‐vis optical titration. For CYP121A1, the typical low‐spin ligand free spectrum shows five characteristic peaks; a protein peak from the aromatic amino acid residues at 280 nm, a Soret peak at 416.5 nm, the β band at 538 nm, the α band at 565 nm, and small charge transfer band at ∼650 nm owing to a residual amount of high‐spin heme iron. A heme iron low‐spin (LS) to high‐spin (HS) type I shift occurs as cYY binds to CYP121A1, with the Soret band shifting from 416.5 to 393 nm (the cYY *K*_D_ is 5.82±0.16 μM^13^). Other features of note in the spectral titration are apparent isosbestic points at approximately 420 nm and 492 nm, small red shifts in the alpha and beta band maxima at ∼572 and 547 nm, and loss of the small high‐spin signal at ∼650 nm. Coordination binding of azoles to the CYP121A1 heme iron results in type II (red) spectral shifts of the Soret peak to wavelengths typically between ∼418–424 nm (according to the specific azole and whether it binds directly to the heme iron or via a retained water ligand to the heme iron), accompanied by spectral changes in both the α and β bands (e.g. **10f**, Figure 4).^19^

In Series 1, replacing the phenyl ring of the lead imidazole compound (**1**) or triazole compound (**2**) with a pyridyl ring resulted in decreased binding affinity compared with the lead phenyl imidazole (**1**) and triazole (**2**) compounds (**1** *K*_D_=2.6±0.2 μM *c.f*. **10 a** *K*_D_=60.5±10.5 μM and **10 b** *K*_D_=53.6±6.8 μM ; **2** *K*_D_=73.5±0.7 μM *c.f*. **11 a** *K*_D_=115.3±27.0 μM and **11 b** *K*_D_=249.7±51.6 μM) (Table 1).

**Table 1:** *K*_D_ and MIC values against *M. tuberculosis* H37Rv.

Compound	*K*_D_ (μM)	Soret peak shift (nm)	MIC (μg/mL)
**10 a**	60.5±10.5	416.5 to 419	12.5
**10 b**	53.6±6.8	416.5 to 421	12.5
**10 c**	18.3±3.4	416.5 to 420	1.562
**10 d**	53.7±5.9	416.5 to 420	0.781
**10 e**	22.5±3.5	416.5 to 423	1.562
**10 f**	0.22±0.01	416.5 to 423.5	1.562
**10 g**	4.81±0.31	416.5 to 419.5	1.562
**10 h**	2.91±0.14	416.5 to 423.5	3.125
**10 i**	10.1±0.3	416.5 to 422.5	3.125
**10 j**	3.31±0.05	416.5 to 423.5	25
**11 a**	115.3±27.0	416.5 to 417.5	>100
**11 b**	249.7±51.6	416.5 to 417.5	>100
**13 a**	83.2±3.9	416.5 to 423.5	100
**13 b**	85.6±12.4	416.5 to 417.5	>100
**14 a**	218.6±13.6	416.5 to 419.5	>100
**14b**	39.0±8.2	416.5 to 417.5	>100
**23**	34.5±4.8	416.5 to 418	>100
**24**	105±7	416.5 to 418	>100
**25**	22.9±0.2	416.5 to 418.5	25
**26**	98.3±7.4	416.5 to 416	100
**1**	2.6±0.2	416.5 to 424	12.5
**2**	73.5±0.7	416.5 to 422.5	50
Fluconazole	8.6±0.2		>100
Clotrimazole	0.07±0.01		20
cYY	5.82±0.16		–

For the extended pyridyl compounds (**13** and **14**), low binding affinity was generally observed, with the exception of **14 b** which displayed moderate binding affinity (*K*_D_=39.0±8.2 μM). The series 2 imidazole pyrazole regioisomers **23** and **25** displayed moderate binding affinity (*K*_D_=34.5±4.8 μM and 22.9±0.2 μM, respectively). However, the triazole pyrazole regioisomers **24** and **26** displayed weak binding affinity (*K*_D_=105±7 μM and 98.3±7.4 μM, respectively) (Table 1).

Series 3 biaryl derivatives with branched butyl substitutions (**10 f** and **10 g**), the biphenyl substitution (**10 h**) and the indole substitution (**10 j**) gave the highest affinity for CYP121A1, having *K*_D_ values <5 μM (*K*_D_=0.22±0.01 μM, 4.81±0.31 μM, 2.91±0.14 μM and 3.31±0.05 μM, respectively) (Table 1). The aryl derivatives with shorter alkyl substitutions, 4‐ethyl (**10 c**), 4‐propyl (**10 d**), 4‐isopropyl (**10 e**) and benzo [*d*][1,3] dioxole (**10 i**) had moderate binding affinity (*K*_D_=18.3±3.4 μM, 53.7±5.9 μM, 22.5±3.5 μM, and 10.1±0.3 μM, respectively). The values were compared with the *K*_D_ values of fluconazole (8.6±0.2 μM) and clotrimazole (0.07±0.01 μM).^19^

Compounds **10 e**, **10 f**, **10 h**, **10 i**, **10 j** and **13 a** induce the most extensive Soret absorbance shifts, with the Soret maxima shifting by 6.5 nm (in the case of **10 e**) and by 7 nm (in the case of **10 f**, **10 h**, **10 j** and **13 a**). The shifts observed are comparable with those for azole antifungal drugs (clotrimazole, econazole, fluconazole, ketoconazole and miconazole), which bind tightly to *Mtb* CYP121A1, inducing a Soret peak shift to between 421–423.5 nm.^20^ Less extensive Soret red shifts were observed for imidazole pyrazole regioisomers (**23** and **25**), the triazole pyrazole regioisomers (**24** and **26**), the 4‐ethylbenzene (**10 c**), 4‐propylbenzene (**10 d**) and 4‐*tert*‐butylbenzene (**10 g**) and the Series 1 3‐pyridyl and 4‐pyridyl (**10 a** and **10 b**) imidazole derivatives on binding to the CYP121A1 heme iron.

### 2.3 MIC Determination Against *Mycobacterium Tuberculosis*

The derivatives were screened against *M. tuberculosis* H37Rv by the REMA (Resazurin Microtiter Assay) method.^21^ In Series 1 only the imidazole pyridyl derivatives displayed antimicrobial activity (**10 a** and **10 b**, MIC=12.5 μg/mL), retaining the inhibitory activity observed for the phenyl imidazole lead compound (**1**).

In Series 2 only the 5‐((1*H*‐imidazol‐1‐yl)methyl)‐1‐(4‐methoxyphenyl)‐3‐methyl‐1*H*‐pyrazole (**23**) displayed antimycobacterial activity with an MIC of 25 μg/mL (Table 1). All the compounds in Series 3 displayed antimycobacterial activity, the most promising inhibitory activity (MIC=0.781–1.562 μg/mL) was observed for the alkyl substituted aryl derivatives (**10 c**–**10 g**). Good inhibitory activity was also obtained for biphenyl (**10 h**, MIC=3.125 μg/mL) and benzo[*d*][1,3]dioxole (**10 i**, MIC=3.125 μg/mL) derivatives, with the least active in Series 3 being the 2‐chloro‐1*H*‐indole derivative (**10 j**, MIC=25 μg/mL).

**Figure 5:**
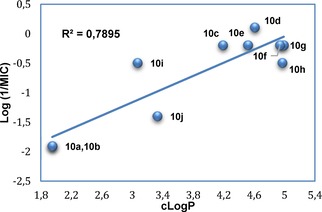
Hansch analysis illustrating correlation between MIC and calculated LogP^23^ values for the Series 1 and 3 imidazole compounds (**10**).

Hansch analysis (Figure 5) showed a clear correlation between the lipophilicity of the compounds and MIC, as previously observed for the pyrazole lead compounds.^13^ This increased lipophilicity may facilitate increased drug uptake across the *Mtb* lipid‐rich cell wall resulting in enhanced antimycobacterial activity. The more lipophilic compounds **10 c**–**10 g** (cLogP 4.19–4.99) showed the best antimycobacterial activity (MIC=0.781–1.562 μg/mL), while the more polar pyridyl (**10 a** and **10 b**) (cLogP 1.95) and 2‐chloro‐1*H*‐indole (**10 j**) (cLogP 3.33) compounds had lower antimycobacterial activity (MIC=12.5–25 μg/mL).

### 2.4 Molecular Modelling

**Figure 6:**
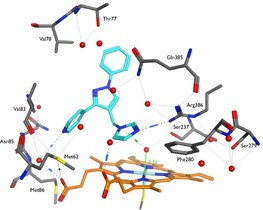
Series 1 pyridyl compound **10 a** in CYP121A1 active site. The imidazole ring interacts with the heme iron through an interstitial water molecule bonded with Ser237 and a direct hydrogen bond with Arg386. Additional binding interactions through the pyridine nitrogen are observed via a water molecule with Met62, Met86 and Val83.

The Molecular Operating Environment (MOE) program^22^ was used to perform molecular docking and was found to closely replicate the position and binding interactions of cYY and fluconazole, as observed in the crystal structures PDB 3G5H and PDB 2IJ7, respectively. The 4‐ and 3‐pyridyl derivatives (**10/11 a** and **10/11 b**) both interacted with the heme iron through an interstitial water molecule bonded with Ser237, and the 4‐pyridyl (**10 a**) also formed a direct hydrogen bond with Arg386 (e. g. **10 a**, Figure 6). Both pyridyl imidazoles (**10**) and triazoles (**11**) formed additional binding interactions through the pyridine nitrogen, for the 3‐pyridyl via a water molecule with Met62 and for the 4‐pyridyl via a water molecule with Met62, Met86 and Val83 (Figure 6).

The imidazole derivatives (**13**) with the 5‐atom linker between the imidazole and the pyrazole ring were found to form more interactions with the CYP121A1 active site compared with the 4‐pyridyl derivatives (**14**) with the 3‐atom linker between the 4‐pyridyl and pyrazole ring. However, the extended pyridine compounds (**13** and **14**) were found to form numerous docking conformations owing to the increased flexibility of the extended linker between the heme‐bonding group, imidazole for **13** and 4‐pyridyl for **14**. Series 2 imidazole (**23** and **25**) and triazole (**24** and **26**) derivatives, with the smaller methyl replacing a phenyl ring, were found to interact with the heme via an interstitial water molecule bound to Ser237, and for imidazole **23** a direct hydrogen bond interaction with Arg386 was formed. Additional hydrophobic interactions were observed with hydrophobic amino acid residues including Thr77, Val78, Val82, Val83 and Met86.

**Figure 7:**
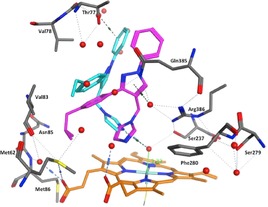
Overlapped images of Series 3 ethyl (**10 c**) and isobutyl (**10 f**) derivatives. The ethyl imidazole (**10 c**, cyan) interacts through the imidazole with the heme indirectly via an interstitial water molecule bonded to Ser237, and through the phenyl ring via an interstitial water molecule bonded to Thr77. The isobutyl imidazole (**10 f**, magenta) interacts with the heme in a similar manner as observed for **10 c**, but forms an additional interaction through the pyrazole ring and occupies the binding site more fully.

The ethyl (**10 c**) and propyl (**10 d**) imidazoles were found to bind in the same manner through interactions with the heme iron via an interstitial water molecule linked to Ser237 and with the phenyl ring interacting with Thr77 via a water molecule. The isopropyl (**10 e**) and isobutyl (**10 f**) imidazoles interacted with the heme in the same manner as described for **10 c** and **10 d**, but also formed an additional interaction between the pyrazole ring either with Thr77 via a water molecule in the case of **10 e**, or with Gln385 and Arg386 via a water molecule for **10 f**. The *tert*‐butyl imidazole (**10 g**) and the biphenyl imidazole (**10 h**) both formed a direct hydrogen bond with Arg386 and interacted with the heme through an interstitial water molecule bonded with Ser237. The phenyl ring of the biphenyl imidazole (**10 h**) also formed an interaction with Thr77 via a water molecule. The positioning of the additional binding interactions of the branched alkyl and biphenyl imidazoles (**10 e**–**10 h)** resulted in a better filling of the binding pocket (Figure 7), especially in the case of **10 f** and **10 g**, and therefore these compounds would be predicted to be more efficient at blocking binding of the cYY natural substrate.

The heterobiaryl benzo[*d*][1,3]dioxole (**10 i**) and 2‐chloro‐1*H*‐indole (**10 j**) derivatives showed binding of the imidazole with the heme via an interstitial water molecule through Ser237, and additional H‐bond interactions via Thr77, Arg386 and Gln385. The more extended pyrazole derivatives (**10 f**, **10 g**, **10 h**, **10 i** and **10 j**) either formed additional binding interactions and/or more completely blocked the active site by occupying a similar binding site as the natural substrate cYY and fluconazole, resulting in improved binding affinity.

### 2.5 X‐Ray Crystallographic Studies

**Figure 8:**
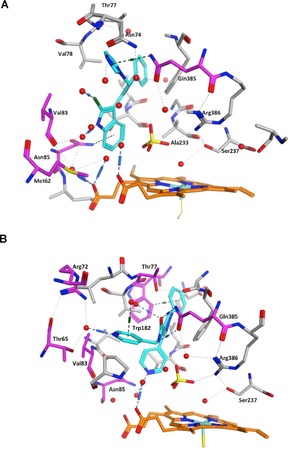
X‐ray crystal structures of (**A**) **10 j** (PDB 6GEO) and (**B**) **14 a** (PDB 6GEQ) binding to CYP121A1. The compounds are shown in cyan and the haem in orange. Water molecules are illustrated with small red spheres and amino acids are presented either in light grey or, for amino acids that form direct or indirect H‐bonding interactions with the inhibitor compound, in magenta. The sulfate molecule from the crystallography process is seen above the heme and forms H‐bond interactions with Arg386 and indirectly with Ser237.

Two compounds were successful co‐crystallized with CYP121A1, **10 j** and **14 a** (PDB 6GEO and 6GEQ respectively). Both crystals contained two molecules of sulfate resulting from the crystallography process, one of which is positioned above the heme (Figure 8).

The indole derivative **10 j** forms an arene‐H interaction between the imidazole ring and Gln385, and between the indole benzene ring and Asn85. The indole NH and Cl act as H‐donors with two water molecules and the imidazole N acts as a H‐acceptor with a water molecule. The pyrrole aryl ring is positioned to form a π‐π interaction with Phe168 (Figure 8 A). The extended 4‐pyridyl derivative **14 a** makes a H‐bond interaction between the carbonyl oxygen and Gln385, and interacts indirectly with Thr77. Both 4‐pyridyl groups form indirect H‐bond interactions: the amide pyridyl with Thr65 and Arg72, and the pyrrole pyridyl with Val83 and Asn65. The benzene ring forms an arene‐H interaction with Trp182 (Figure 8B).

**Figure 9:**
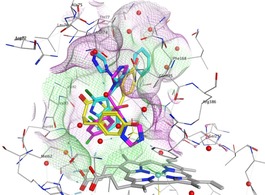
Alignment of the crystal structures of CYP121A1 co‐crystallized with **10 j** (cyan) (PDB 6GEO), fluconazole (magenta) (PDB 2IJ7) and cYY (yellow) (PDB 3G5H) with comparable positioning observed.

Alignment of the crystal structure of **10 j** co‐crystallized with CYP121A1 (PDB 6GEO) with the crystal structures of CYP121A1 co‐crystallized with fluconazole (PDB 2IJ7) and cYY (PDB 3G5H) showed a comparable position of all three structures in the enzyme (Figure 9). All three compounds are positioned to form a π‐π interaction with Phe168 and interact either directly or indirectly with amino acids Met62, Val83, Asn85. In particular, H‐bonding interactions with Gly385 (**10 j** and fluconazole) or Arg386 (cYY) would appear to effectively block access to the active site.

## 3 Conclusions

For optimal binding interactions, inhibitors that effectively fill the CYP121A1 binding site and exhibit strong binding affinity are those that should most effectively inhibit CYP121A1 substrate (cYY) binding. This is observed for the branched alkyl derivatives **10 f** and **10 g**, and for the biphenyl **10 h**. Compound flexibility also has a notable effect, with the extended pyridine derivatives **13** and **14** displaying very weak binding affinity. Lipophilicity is a major contributor to antimycobacterial activity, as shown by Hansch analysis (Figure 5). The challenge in designing a compound with both good binding affinity and antimycobacterial activity is to combine the most favourable properties for each component. The physicochemical properties of the prepared compounds and reference compounds (cYY, fluconazole and clotrimazole) were calculated, the cLogP was determined using Crippen's fragmentation^23^ and the number of H‐bond acceptors (nON), H‐bond donors (nOHNH) rotatable bonds (nrot), along with the molecular volume (MV) and topological polar surface area (TPSA) were calculated using Molinspiration software.^24^ Those compounds with cLogP >4 and TPSA <40 Å^2^ had optimal antimycobacterial activity (shaded green, Table 2), while the compounds with 5–6 rotatable bonds, 4–5 H‐bond acceptors, no H‐bond donors and molecular volume >340 Å^3^ had optimal binding affinity (shaded red, Table 2).

**Table 2:** Physiocochemical properties of Series 1, 2 and 3.

Compound	cLogP	nON/ nOHNH	nrot	MV (Å^3^)	TPSA (Å ^2^)
**10 a/10 b**	1.95	5/0	4	273.34	48.54
**10 c^[a]^**	4.19	4/0	5	310.86	35.65
**10 d^[a]^**	4.61	4/0	6	327.66	35.65
**10 e^[a]^**	4.52	4/0	5	327.44	35.65
**10 f^[a,b]^**	4.94	4/0	6	344.24	35.65
**10 g^[a,b]^**	4.99	4/0	5	343.68	35.65
**10 h^[b]^**	4.97	4/0	5	348.90	35.65
**10 i**	3.07	6/0	4	301.42	54.12
**10 j^[b]^**	2.68	5/1	4	306.47	51.44
**11 a/11 b**	1.46	6/0	4	269.18	61.44
**13 a/b**	1.26	7/1	7	338.33	77.64
**14 a/b**	1.44	7/1	5	319.36	72.71
**23**	1.0	5/0	4	248.19	44.89
**24**	0.5	6/0	4	244.03	57.78
**25**	0.84	5/0	4	248.19	44.89
**26**	0.35	6/0	4	244.03	57.78
cYY	1.26	6/4	4	290.68	98.65
Fluconazole	0.87	7/1	5	248.96	81.66
Clotrimazole	5.97	2/0	4	309.52	17.83

[a] Optimal antimycobacterial activity (MIC). [b] Optimal binding affinity (*K*_D_). nON=H‐bond acceptor; nOHNH=H‐bond donor; nrot=number of rotatable bonds; MV=molecular volume; TPSA=topological polar surface area.

Therefore, the optimal combined physicochemical properties for antimycobacterial activity and binding affinity (shaded green and red, Table 2) identified Series 3 isobutyl (**10 f**) and *tert*‐butyl derivatives (**10 g**) as fulfilling these requirements. These and other high affinity compounds generated in this study will undergo further testing for their ability to kill pathogenic strains of *M. tuberculosis*.

## Experimental Section

### Chemistry

All reagents and solvents were of general purpose or analytical grade and purchased from Sigma‐Aldrich Ltd, Fisher Scientific, Fluka and Acros. ^1^H and ^13^C NMR spectra were recorded with a Bruker Avance DPX500 spectrometer operating at 500 and 125 MHz, with Me_4_Si as internal standard. Elemental analysis was performed by MEDAC Ltd (Chobham, Surrey, UK). High resolution mass spectra (HRMS) were determined at the EPSRC National Mass Spectrometry Facility at Swansea University and Medac Ltd (Chobham, Surrey, UK), using ESI (Electrospray Ionisation) in positive and negative modes, and a TOF (Time‐of‐Flight) analyser. Flash column chromatography was performed with silica gel 60 (230–400 mesh) (Merck) and TLC was carried out on precoated silica plates (kiesel gel 60 F_254_, BDH). Compounds were visualised by illumination under UV light (254 nm) or by the use of potassium permanganate stain followed by heating. Melting points were determined on an electrothermal instrument and are uncorrected. All solvents were dried prior to use and stored over 4 Å molecular sieves, under nitrogen. All the compounds were ≥95 % pure.

The following compounds were prepared as previously described: hydrazines **6 a** and **6 b**,^14^ **6 h** 23 and **6 j**;^25^ aldehydes **7 a** and **7 b**,^14^ **7 h** 26 and **7 j**;^25^ alcohol **8 h**;^26^ chloride **9 h**;^26^ carboxylic acids (**12 a**) and (**12 b**);^14^ ethyl 1‐(4‐methoxyphenyl)‐5‐methyl‐1*H*‐pyrazole‐3‐carboxylate (**17**) and ethyl 1‐(4‐methoxyphenyl)‐3‐methyl‐1*H*‐pyrazole‐5‐carboxylate (**18**).^18^ All compounds were more than 95 % pure.

### General Method for the Preparation of Hydrazine Derivatives (6)

To a solution of the acetyl reagent (**4**) (5 mmol) in ethanol (20 mL), was added acetic acid (3.3 mmol) with continuous stirring at room temperature. After 5 min, phenylhydrazine (**5**) (5.5 mmol) was added dropwise with further stirring for 15 min, then refluxed at 80 °C for 1 h. The reaction was left to cool, concentrated under reduced pressure, wrapped with aluminium foil and left in the freezer overnight. The precipitate formed was filtered, washed with cold ethanol, dried *in vacuo* and used immediately in subsequent reactions without further purification.

### 1‐(1‐(4‐Ethylphenyl)ethylidene)‐2‐phenylhydrazine (6 c)

Prepared from 4‐ethylacetophenone (2 mL, 13.39 mmol). Product obtained as a yellow solid, yield 3.03 g (95 %). M.p. 82–84 °C. TLC (1 : 1 petroleum ether/EtOAc), R*f*=0.94. ^1^H NMR (DMSO‐d_6_): δ 9.19 (s, 1H, NH), 7.70 (d, *J*=8.2 Hz, 2H, Ar), 7.23 (m, 6H, Ar), 6.75 (m, 1H, para‐Ar), 2.61 (q, *J*=7.6 Hz, 2H, CH_2_), 2.24 (s, 3H, CH_3_), 1.19 (t, *J*=7.6 Hz, 3H, CH_3_). ^13^C NMR (DMSO‐d_6_): δ 146.7 (C=N), 143.6 (C, Ar), 141.2 (C, Ar), 137.3 (C, Ar), 129.3 (2×CH, Ar), 128.1 (2×CH, Ar), 125.7 (2×CH, Ar), 119.2 (CH, para‐Ar), 113.2 (2×CH, Ar), 28.3 (CH_2_), 16.0 (CH_3_), 13.3 (CH_3_).

### (1‐(1‐(4‐Propylphenyl)ethylidene)‐2‐phenylhydrazine (6 d)

Prepared from 4‐propylacetophenone (1 mL, 6.04 mmol). Product obtained as an orange crystalline solid, yield 1.43 g (92 %). M.p. 68–70 °C. TLC (2 : 1 petroleum ether/EtOAc), R*f*=0.90. ^1^H NMR (DMSO‐d_6_): δ 9.19 (s, 1H, NH), 7.70 (d, *J*=7.9 Hz, 2H, Ar), 7.23 (m, 6H, Ar), 7.09 (t, *J*=7.7 Hz, 1H, para‐Ar), 2.56 (t, *J*=7.5 Hz, 2H, CH_2_), 2.24 (s, 3H, CH_3_), 1.60 (sext, *J*=7.3 Hz, 2H, CH_2_), 0.90 (t, *J*=7.3 Hz, 3H, CH_3_). ^13^C NMR (DMSO‐d_6_): δ 146.7 (C=N), 142.0 (C, Ar), 141.1 (C, Ar), 137.3 (C, Ar), 128.7 (2×CH_,_ Ar), 127.7 (2×CH, Ar), 125.6 (2×CH, Ar), 119.2 (1×CH_,_ para‐Ar), 113.2 (2×CH_,_ Ar), 37.4 (CH_2_), 24.8 (CH_2_), 14.6 (CH_3_), 13.3 (CH_3_).

### 1‐(1‐(4‐Isopropylphenyl)ethylidene)‐2‐phenylhydrazine (6 e)

Prepared from 4‐isopropylacetophenone (2 mL, 11.95 mmol). Product obtained as an orange crystalline solid, yield 2.5 g (80 %). M.p. 50–52 °C. TLC (1 : 1 petroleum ether/EtOAc), R*f*=0.93. ^1^H NMR (DMSO‐d_6_): δ 9.19 (s, 1H, NH), 7.70 (d, *J*=7.2 Hz, 2H, Ar), 7.25 (d, *J*=7.7 Hz, 2H, Ar), 7.21 (m, 4H, Ar), 6.75 (t, *J*=6.7 Hz, 1H, para‐Ar), 2.90 (m, 1H, CH), 2.24 (s, 3H, CH_3_), 1.22 (d, *J*=7.1 Hz, 6H, 2×CH_3_). ^13^C NMR (DMSO‐d_6_): δ 148.3 (C=N), 146.7 (C, Ar), 141.1 (C, Ar), 137.5 (C, Ar), 129.3 (2×CH, Ar), 126.6 (2×CH, Ar), 125.7 (2×CH, Ar), 119.1 (CH, para‐Ar), 113.2 (2×CH, Ar), 33.6 (CH), 24.3 (2×CH_3_), 13.3 (CH_3_).

### 1‐(1‐(4‐Isobutylphenyl)ethylidene)‐2‐phenylhydrazine (6 f)

Prepared from 4‐isobutylacetophenone (5 mL, 27.00 mmol). Product obtained as an orange solid, yield 6.99 g (97 %). M.p. 58–60 °C. TLC (3 : 1 petroleum ether/EtOAc), R*f*=0.71. ^1^H NMR (DMSO‐d_6_): δ 9.18 (s, 1H, NH), 7.69 (d, *J*=7.7 Hz, 2H, Ar), 7.22 (m, 4H, Ar), 7.16 (d, *J*=7.7 Hz, 2H, Ar), 6.75 (t, *J*=7.3 Hz, 1H, para‐Ar), 2.44 (d, *J*=7.6 Hz, 2H, CH_2_), 2.23 (s, 3H, CH_3_), 1.83 (m, 1H, CH), 0.86 (d, *J*=7.6 Hz, 6H, 2×CH_3_). ^13^C NMR (DMSO‐d_6_): δ 146.6 (C=N), 141.2 (C, Ar), 141.1 (C, Ar), 137.3 (C, Ar), 129.4 (2×CH, Ar), 129.3 (2×CH, Ar), 125.4 (2×CH, Ar), 119.2 (CH, para‐Ar), 113.2 (2×CH, Ar), 44.8 (CH_2_), 30.0 (CH_3_), 22.6 (CH), 13.4 (2×CH_3_).

### 1‐(1‐(4‐(*Tert*‐butyl)phenyl)ethylidene)‐2‐phenylhydrazine (6 g)

Prepared from 4‐(*tert*‐butyl)acetophenone (3 mL, 16.4 mmol). Product obtained as an orange crystalline solid, yield 4.2 g (97 %). M.p. 66–68 °C. TLC (1 : 1 petroleum ether/EtOAc), R*f*=0.92. ^1^H NMR (DMSO‐d_6_): δ 9.25 (s, 1H, NH), 7.75 (d, *J*=8.4 Hz, 2H, Ar), 7.40 (d, *J*=8.4 Hz, 2H, Ar), 7.31 (m, 4H, Ar), 7.13 (t, *J*=7.7 Hz, 1H, para‐Ar), 2.27 (s, 3H, CH_3_), 1.31 (s, 9H, 3×CH_3_). ^13^C NMR (DMSO‐d_6_): *δ* 150.4 (C=N), 146.7 (C, Ar), 141.1 (C, Ar), 137.1 (C, Ar), 129.3 (2×CH, Ar), 129.0 (2×CH, Ar), 125.4 (2×CH, Ar), 119.2 (CH, para‐Ar), 113.3 (2×CH, Ar), 34.7 (C(CH_3_)_3_), 31.5 (3×CH_3_), 13.3 (CH_3_).

### 1‐(1‐(Benzo[d][1,3]dioxol‐5‐yl)ethylidene)‐2‐phenylhydrazine (6 i)

Prepared from 3',4'‐(methylenedioxy)acetophenone (3.00 g, 18.27 mmol). Product obtained as a yellow solid, yield 3.90 g (84 %). M.p. 66–68 °C. TLC (1 : 1 petroleum ether/EtOAc), R*f*=0.88. ^1^H NMR (DMSO‐d_6_): δ 9.14 (s, 1H, NH), 7.40 (s, 1H, benzo[*d*][1,3]dioxole), 7.23 (d, *J*=1.6 Hz, 1H, benzo[*d*][1,3]dioxole), 7.21 (m, 4H, Ar), 6.92 (d, *J*=8.6 Hz, 1H, benzo[*d*][1,3]dioxole), 6.74 (t, *J*=4.1 Hz, 1H, para‐Ar), 6.04 (s, 2H, benzo[*d*][1,3]dioxole), 2.21 (s, 3H, CH_3_). ^13^C NMR (DMSO‐d_6_): *δ* 148.0 (C=N), 147.5 (C, benzo[*d*][1,3]dioxole), 146.7 (C, benzo[*d*][1,3]dioxole), 140.9 (C, Ar), 134.3 (C, Ar), 134.3 (C, Ar), 129.3 (2×CH, Ar), 119.7 (CH, para‐Ar), 119.1 (CH, benzo[*d*][1,3]dioxole), 113.2 (CH, benzo[*d*][1,3]dioxole), 108.3 (2×CH, Ar), 105.6 (C, benzo[*d*][1,3]dioxole), 101.5 (CH_2_, benzo[*d*][1,3]dioxole), 13.5 (CH_3_).

### General Method for the Preparation of Aldehyde Derivatives (7)

Phosphorus (V) oxychloride (15 mmol) was added dropwise to an ice‐cooled solution of hydrazine (**6**) (5 mmol) in dry DMF (50 mL). The reaction mixture was allowed to reach room temperature, and then heated at 60 °C for 4 h with continuous stirring. The resulting mixture was poured onto crushed ice (50 mL), brought to pH 8.0 using 10 % aqueous NaOH (≈50 mL), and left overnight in the freezer. The resulting precipitate was extracted with EtOAc (100 mL) and the organic layer was washed with brine (5×50 mL), while the aqueous phase was re‐extracted with EtOAc (3×50 mL). The combined organic fractions were washed with brine (3×50 mL), dried (MgSO_4_) and evaporated *in vacuo* to obtain the crude product, which was purified by gradient column chromatography.

### 3‐(4‐Ethylphenyl)‐1‐phenyl‐1*H*‐pyrazole‐4‐carbaldehyde (7 c)

Prepared from 1‐(1‐(4‐ethylphenyl)ethylidene)‐2‐phenylhydrazine (**6 c**) (2.5 g, 10.48 mmol). The product was eluted with petroleum ether – EtOAc 85 : 15 v/v to give the product as a buff solid, yield 1.76 g (61 %). M.p. 112–114 °C. TLC (1 : 1 petroleum ether/EtOAc), R*f*=0.88. ^1^H NMR (DMSO‐d_6_): δ 9.99 (s, 1H, CHO), 9.31 (s, 1H, pyrazole), 8.00 (d, *J*=7.6 Hz, 2H, Ar), 7.86 (d, *J*=8.1 Hz, 2H, Ar), 7.58 (t, *J*=7.5 Hz, 2H, Ar), 7.43 (t, *J*=7.5 Hz, 1H, para‐Ar), 7.36 (d, *J*=8.3 Hz, 2H, Ar), 2.69 (q, *J*=7.6 Hz, 2H, CH_2_), 1.24 (t, *J*=7.6 Hz, 3H, CH_3_). ^13^C NMR (DMSO‐d_6_): δ 185.0 (CHO), 153.4 (C, pyrazole), 145.4 (C, Ar), 139.1 (C, Ar), 135.1 (CH, pyrazole), 130.1 (2×CH, Ar), 129.1 (2×CH, Ar), 128.5 (2×CH, Ar), 128.3 (CH, para‐Ar), 122.7 (C, pyrazole), 122.6 (C, Ar), 119.8 (2×CH, Ar), 28.4 (CH_2_), 16.0 (CH_3_). Anal. Calcd for C_18_H_16_N_2_O (276.3372): C, 78.24; H, 5.84; N, 10.13. Found: C, 78.11; H, 5.84; N, 10.13.

### 1‐Phenyl‐3‐(4‐propylphenyl)‐1*H*‐pyrazole‐4‐carbaldehyde (7 d)

Prepared from 1‐(1‐(4‐propylphenyl)ethylidene)‐2‐phenylhydrazine (**6 d**) (1.20 g, 4.75 mmol). The product was eluted with petroleum ether – EtOAc 85 : 15 v/v to give the product as a white solid, yield 0.91 g (66 %). M.p. 86–88 °C. TLC (3 : 1 petroleum ether/EtOAc), R*f*=0.68. ^1^H NMR (DMSO‐d_6_): δ 9.99 (s, 1H, CHO), 9.31 (s, 1H, pyrazole), 8.00 ( d, *J*=7.6 Hz, 2H, Ar), 7.85 (d, *J*=7.8 Hz, 2H, Ar), 7.58 (t, *J*=7.5 Hz, 2H, Ar), 7.43 (t, *J*=7.5 Hz, 1H, para‐Ar), 7.34 (d, *J*=7.8 Hz, 2H, Ar), 2.63 (t, *J*=7.4 Hz, 2H, CH_2_), 1.65 (sext, *J*=7.3 Hz, 2H, CH_2_), 0.93 (t, *J*=7.4 Hz, 3H, CH_3_). ^13^C NMR (DMSO‐d_6_): δ 185.0 (CHO), 153.2 (C, pyrazole), 143.9 (C, Ar), 139.1 (C, Ar), 135.2 (CH, pyrazole), 130.2 (2×CH, Ar), 130.1 (C, Ar), 129.2 (2×CH, Ar), 129.1 (2×CH, Ar), 128.2 (CH, para‐Ar), 122.6 (C, pyrazole), 119.7 (2×CH, Ar), 37.5 (CH_2_), 24.5 (CH_2_), 14.1 (CH_3_). Anal. Calcd for C_19_H_18_N_2_O (290.364): C, 78.59; H, 6.25; N, 9.64. Found: C, 78.37; H, 6.36; N, 9.68.

### 3‐(4‐(Isopropylphenyl)‐1‐phenyl‐1*H*‐pyrazole‐4‐carbaldehyde (7 e)

Prepared from 1‐(1‐(4‐isopropylphenyl)ethylidene)‐2‐phenylhydrazine (**6 e**) (2.30 g, 9.24 mmol). The product was eluted with petroleum ether – EtOAc 85 : 15 v/v to give the product as a white solid, yield 2.30 g (78 %). M.p. 114–116 °C. TLC (1 : 1 petroleum ether/EtOAc), R*f*=0.83. ^1^H NMR (DMSO‐d_6_): δ 9.99 (s, 1H, CHO), 9.31 (s, 1H, pyrazole), 8.01 (d, *J*=7.6 Hz, 2H, Ar), 7.86 (d, *J*=8.1 Hz, 2H, Ar), 7.58 (t, *J*=8.2 Hz, 2H, Ar), 7.43 (t, *J*=8.3 Hz, 1H, para‐Ar), 7.39 (d, *J*=8.2 Hz, 2H, Ar), 2.97 (sept, *J*=6.8 Hz, 1H, CH), 1.26 (d, *J*=6.9 Hz, 6H, 2×CH_3_). ^13^C NMR (DMSO‐d_6_): δ 185.2 (CHO), 153.2 (C, pyrazole), 150.1 (C, Ar), 139.1 (C, Ar), 135.2 (CH, pyrazole), 130.2 (2×CH, Ar), 129.3 (C, Ar), 129.2 (2×CH, Ar), 128.2 (CH, para‐Ar), 127.0 (2×CH, Ar), 122.6 (1 C, pyrazole), 119.7 (2×CH, Ar), 33.8 (CH), 24.3 (2×CH_3_). Anal. Calcd for C_19_H_18_N_2_O (290.364): C, 78.59; H, 6.25; N, 9.64. Found: C, 78.42; H, 6.15; N, 9.58.

### 3‐(4‐Isobutylphenyl)‐1‐phenyl‐1*H*‐pyrazole‐4‐carbaldehyde (7 f)

Prepared from 1‐(1‐(4‐isobutylphenyl)ethylidene)‐2‐phenylhydrazine (**6 f**) (6.00 g, 22.52 mmol). The product was eluted with petroleum ether – EtOAc 4 : 1 v/v to give the product as a yellow oil, yield 4.82 g (70 %). TLC (3 : 1 petroleum ether/EtOAc), R*f*=0.38. ^1^H NMR (DMSO‐d_6_): δ 9.98 (s, 1H, CHO), 9.27 (s, 1H, pyrazole), 7.97 (d, *J*=7.8 Hz, 2H, Ar), 7.84 (d, *J*=7.7 Hz, 2H, Ar), 7.57 (t, *J*=7.7 Hz, 2H, Ar), 7.42 (t, *J*=7.6 Hz, 1H, para‐Ar), 7.28 (d, *J*=7.8 Hz, 2H, Ar), 2.51 (d, *J*=6.8 Hz, 2H, CH_2_), 1.88 (m, 1H, CH), 0.88 (d, *J*=6.7 Hz, 6H, 2×CH_3_). ^13^C NMR (DMSO‐d_6_): δ 185.0 (CHO), 153.1 (C, Ar), 142.8 (C, pyrazole), 139.1 (C, Ar), 135.4 (CH, pyrazole), 130.2 (2×CH, Ar), 129.6 (2×CH, Ar), 129.2 (C, Ar), 128.9 (2×CH, Ar), 128.2 (CH, para‐Ar), 122.6 (C, pyrazole), 119.7 (2×CH, Ar), 44.8 (CH_2_), 30.1 (CH), 22.6 (2×CH_3_). [ESI‐HRMS] calculated for C_20_H_21_N_2_O: 305.1648 [M+H]^+^. Found: 305.1651 [M+H]^+^.

### 3‐(4‐(Tert‐butyl)phenyl)‐1‐phenyl‐1*H*‐pyrazole‐4‐carbaldehyde (7 g)

Prepared from 1‐(1‐(4‐(*tert*‐butyl)phenyl)ethylidene)‐2‐phenylhydrazine (**6 g**) (3.50 g, 13.13 mmol). The product was eluted with petroleum ether – EtOAc 4 : 1 v/v to give the product as a yellow solid, yield 3.24 g (81 %). M.p. 96–98 °C. TLC (3 : 1 petroleum ether/EtOAc), R*f*=0.18. ^1^H NMR (DMSO‐d_6_): δ 10.01 (s, 1H, CHO), 9.30 (s, 1H, pyrazole), 8.00 (d, *J*=7.6 Hz, 2H, Ar), 7.87 (d, *J*=8.3 Hz, 2H, Ar), 7.56 (t, *J*=8.2 Hz, 2H, Ar), 7.51 (d, *J*=8.4 Hz, 2H, Ar), 7.35 (t, *J*=7.6 Hz, 1H, para‐Ar), 1.31 (s, 9H, 3×CH_3_). ^13^C NMR (DMSO‐d_6_): δ 185.1 (CHO), 153.2 (C, Ar), 152.2 (C, pyrazole), 139.1 (C, Ar), 135.1 (CH, pyrazole), 130.2 (2×CH, Ar), 129.0 (C, pyrazole), 128.9 (2×CH, Ar), 128.1 (CH, para‐Ar), 125.8 (2×CH, Ar), 122.6 (C, pyrazole), 119.7 (2×CH, Ar), 34.9 (C(CH_3_)_3_), 31.5 (3×CH_3_). [ESI‐HRMS] calculated for C_20_H_21_N_2_O: 305.1648 [M+H]^+^. Found: 305.1652 [M+H]^+^.

### 3‐(Benzo[d][1,3]dioxol‐5‐yl)‐1‐phenyl‐1*H*‐pyrazole‐4‐carbaldehyde (7 i)

Prepared from1‐(1‐(benzo[*d*][1,3]dioxol‐5‐yl)ethylidene)‐2‐phenylhydrazine (**6 i**) (1.62 g, 6.37 mmol). The product was eluted with petroleum ether – EtOAc 7 : 1 v/v to give the product as a yellow solid, yield 1.50 g (81 %). M.p. 167–169 °C. TLC (1 : 1 petroleum ether/EtOAc), R*f*=0.94. ^1^H NMR (DMSO‐d_6_): δ 9.96 (s, 1H, CHO), 9.31 (s, 1H, pyrazole), 7.99 (d, *J*=7.7 Hz, 2H, Ar), 7.57 (t, *J*=7.6 Hz, 2H, Ar), 7.53 (s, 1H, benzo[*d*][1,3]dioxole), 7.51 (d, *J*=1.6 Hz, 1H, benzo[*d*][1,3]dioxole), 7.43 (t, *J*=7.4 Hz, 1H, para‐Ar), 7.05 (d, *J*=8.6 Hz, 1H, benzo[*d*][1,3]dioxole), 6.11 (s, 2H, benzo[*d*][1,3]dioxole). ^13^C NMR (DMSO‐d_6_): δ 185.0 (CHO), 152.7 (C, pyrazole), 148.6 (C, benzo[*d*][1,3]dioxole), 147.9 (C, benzo[*d*][1,3] dioxole), 139.0 (C, Ar), 135.8 (CH, pyrazole), 130.2 (2×CH, Ar), 128.1 (CH, para‐Ar), 125.6 (C, benzo[*d*][1,3]dioxole), 123.4 (CH, benzo[*d*][1,3]dioxole), 122.4 (C, pyrazole), 119.7 (2×CH, Ar), 109.2 (CH, benzo[*d*][1,3]dioxole), 108.9 (CH, benzo[*d*][1,3]dioxole), 101.8 (CH_2_, benzo[*d*][1,3]dioxole). [ESI‐HRMS] calculated for C_17_H_13_N_2_O_3_: 293.0924 [M+H]^+^. Found: 293.0921 [M+H]^+^.

### General Method for the Preparation of Alcohol Derivatives (8)

To an ice‐cooled solution of carbaldehyde (**7**) (1 mmol) in EtOH (10 mL) was added NaBH_4_ (1 mmol) in portions, then the reaction was then stirred at room temperature for 1 h. The solvent was evaporated and H_2_O (20 mL) was added slowly and the reaction stirred for 30 min. The reaction mixture was extracted with EtOAc (2×25 mL), then the combined organic layers washed with H_2_O (3×25 mL), dried (MgSO_4_) and evaporated under reduced pressure to give the crude alcohol (**17**), which was was further purified by gradient column chromatography.

### (1‐Phenyl‐3‐(pyridin‐4‐yl)‐1*H*‐pyrazol‐4‐yl)methanol (8 a)

Prepared from 1‐phenyl‐3‐(pyridin‐4‐yl)‐1*H*‐pyrazole‐4‐carbaldehyde (**7 a**)^14^ (0.40 g, 1.6 mmol). The product was obtained as a light yellow solid pure enough for use in the following reaction. Yield 0.33 g (82 %). M.p. 120–122 °C. TLC (1 : 4 petroleum ether/EtOAc), R*f*=0.22. ^1^H NMR (DMSO‐d_6_): δ 8.66 (d, *J*=6.0 Hz, 2H, pyridine), 8.58 (s, 1H, pyrazole), 7.90 (d, *J*=7.7 Hz, 2H, Ar), 7.85 (d, *J*=6.0 Hz, 2H, pyridine), 7.54 (d, *J*=7.6 Hz, 2H, Ar), 7.36 (t, *J*=7.4 Hz, 1H, para‐Ar), 5.34 (t, *J*=5.0 Hz, 1H, OH ex), 4.63 (d, *J*=5.0 Hz, 2H, CH_2_). ^13^C NMR (DMSO‐d_6_): δ 150.5 (2×CH, pyridine), 148.2 (C, Ar), 140.5 (C, Ar), 139.8 (C, Ar), 130.1 (2×CH, Ar), 129.8 (CH, Ar), 127.1 (CH, Ar), 123.5 (C, Ar), 122.0 (2×CH, Ar), 118.9 (2×CH, Ar), 54.3 (CH_2_). [ESI‐HRMS] calculated for C_15_H_14_N_3_O: 252.1137 [M+H]^+^. Found: 252.1136 [M+H]^+^.

### (1‐Phenyl‐3‐(pyridin‐3‐yl)‐1*H*‐pyrazol‐4‐yl)methanol (8 b)

Prepared from 1‐phenyl‐3‐(pyridin‐3‐yl)‐1*H*‐pyrazole‐4‐carbaldehyde (**7 b**)^14^ (1.00 g, 4.03 mmol). The product was obtained as a light yellow solid pure enough for use in the following reaction. Yield 0.85 g (85 %). M.p. 111–113 °C. TLC (1 : 4 petroleum ether/EtOAc), R*f*=0.23. ^1^H NMR (DMSO‐d_6_): δ 9.09 (s, 1H, pyridine), 8.60 (d, *J*=6.4 Hz, 1H, pyridine), 8.58 (s, 1H, pyrazole), 8.28 (d, *J*=8.2 Hz, 1H, pyridine), 7.92 (d, *J*=7.6 Hz, 2H, Ar), 7.53 (m, 3H, 1×pyridine, 2×Ar), 7.34 (t, *J*=7.4 Hz, 1H, para‐Ar), 5.27 (t, *J*=5.0 Hz, 1H, OH ex), 4.58 (d, *J*=5.0 Hz, 2H, CH_2_). ^13^C NMR (DMSO‐d_6_): δ 149.0 (CH, pyridine), 148.2 (CH, pyridine), 174.6 (C, Ar), 139.3 (C, pyrazole), 135.1 (CH, pyridine), 128.9 (2×CH, Ar), 128.2 (C, pyrazole), 128.0 (CH, pyridine), 126.5 (2×CH, Ar), 124.0 (CH, para‐Ar), 119.0 (CH, pyrazole), 116.7 (C, pyridine), 54.3 (CH_2_). [ESI‐HRMS] calculated for C_15_H_14_N_3_O: 252.1137 [M+H]^+^. Found: 252.1134 [M+H]^+^.

### (3‐(4‐Ethylphenyl)‐1‐phenyl‐1*H*‐pyrazol‐4‐yl)methanol (8 c)

Prepared from 3‐(4‐ethylphenyl)‐1‐phenyl‐1*H*‐pyrazole‐4‐carbaldehyde (**7 c**) (1.68 g, 6.07 mmol). The product was eluted with petroleum ether – EtOAc 7 : 3 v/v to give the product as a white solid, yield 0.86 g (51 %). M.p. 54–55 °C. TLC (1 : 1 petroleum ether/EtOAc), R*f*=0.71. ^1^H NMR (DMSO‐d_6_): δ 8.48 (s, 1H, pyrazole), 7.89 (d, *J*=7.5 Hz, 2H, Ar), 7.81 (d, *J*=8.0 Hz, 2H, Ar), 7.51 (t, *J*=7.5 Hz, 1H, para‐Ar), 7.30 (m, 4H, Ar), 5.22 (t, *J*=5.1 Hz, 1H, OH ex), 4.56 (d, *J*=4.9 Hz, 2H, CH_2_), 2.65 (q, *J*=7.5 Hz, 2H, CH_2_), 1.22 (t, *J*=7.5 Hz, 3H, CH_3_). ^13^C NMR (DMSO‐d_6_): δ 150.9 (C, pyrazole), 144.0 (C, Ar), 140.0 (C, Ar), 130.9 (C, Ar), 130.0 (2×CH, Ar), 129.1 (CH, para‐Ar), 128.4 (2×CH, Ar), 127.8 (2×CH, Ar), 126.5 (CH, pyrazole), 125.8 (C, pyrazole), 118.5 (2×CH, Ar), 54.6 (CH_2_), 28.4 (CH_2_), 16.0 (CH_3_). [ESI‐HRMS] calculated for C_18_H_19_N_2_O: 279.1497 [M+H]^+^. Found: 279.1503 [M+H]^+^.

### (1‐Phenyl‐3‐(4‐propylphenyl)‐1*H*‐pyrazol‐4‐yl)methanol (8 d)

Prepared from 1‐phenyl‐3‐(4‐propylphenyl)‐1*H*‐pyrazole‐4‐carbaldehyde (**7 d**) (0.80 g, 2.75 mmol). The product was eluted with petroleum ether – EtOAc 7 : 3 v/v to give the product as a yellow solid, yield 0.74 g (92 %). M.p. 86–88 °C. TLC (2 : 1 petroleum ether/EtOAc), R*f*=0.48. ^1^H NMR (DMSO‐d_6_): δ 8.49 (s, 1H, pyrazole), 7.89 (d, *J*=7.6 Hz, 2H, Ar), 7.80 (d, *J*=7.5 Hz, 2H, Ar), 7.51 (t, *J*=7.1 Hz, 1H, para‐Ar), 7.31 (d, *J*=7.3 Hz, 2H, Ar), 7.29 (d, *J*=8.1 Hz, 2H, Ar), 5.18 (t, *J*=5.0 Hz, 1H, OH ex), 4.55 (d, *J*=4.9 Hz, 2H, CH_2_), 2.61 (t, *J*=7.4 Hz, 2H, CH_2_), 1.64 (sext, *J*=7.2 Hz, 2H, CH_2_), 0.93 (t, *J*=7.3 Hz, 3H, CH_3_). ^13^C NMR (DMSO‐d_6_): δ 150.8 (C, pyrazole), 142.3 (C, Ar), 140.0 (C, Ar), 130.9 (C, Ar), 130.0 (2×CH, Ar), 129.1 (2×CH, Ar), 129.0 (CH, para‐Ar), 127.7 (2×CH, Ar), 126.5 (CH, pyrazole), 122.3 (C, pyrazole), 118.5 (2×CH, Ar), 54.6 (CH_2_), 37.5 (CH_2_), 24.5 (CH_2_), 14.1 (CH_3_). [ESI‐HRMS] calculated for C_19_H_21_N_2_O: 293.1654 [M+H]^+^. Found: 293.1654 [M+H]^+^.

### (1‐Phenyl‐3‐(4‐isopropylphenyl)‐1*H*‐pyrazol‐4‐yl)methanol (8 e)

Prepared from 1‐phenyl‐3‐(4‐isopropylphenyl)‐1*H*‐pyrazole‐4‐carbaldehyde (**7 e**) (0.90 g, 3.09 mmol). The product was eluted with petroleum ether – EtOAc 7 : 3 v/v to give the product as a colourless oil, yield 0.76 g (84 %). TLC (1 : 1 petroleum ether/EtOAc), R*f*=0.67. ^1^H NMR (DMSO‐d_6_): δ 8.49 (s, 1H, pyrazole), 7.90 (d, *J*=7.6 Hz, 2H, Ar), 7.82 (d, *J*=8.1 Hz, 2H, Ar), 7.51 (t, *J*=8.3 Hz, 2H, Ar), 7.35 (d, *J*=8.1 Hz, 2H, Ar), 7.31 (m, 1H, para‐Ar), 5.18 (t, *J*=4.9 Hz, 1H, OH ex), 4.57 (d, *J*=4.8 Hz, 2H, CH_2_), 2.94 (sept, *J*=6.8 Hz, 1H, CH), 1.25 (d, *J*=6.9 Hz, 6H, 2×CH_3_). ^13^C NMR (DMSO‐d_6_): δ 150.8 (C, pyrazole), 148.5 (C, Ar), 140.0 (C, Ar), 131.1 (C, Ar), 130.0 (2×CH, Ar), 129.1 (CH, para‐Ar), 127.8 (2×CH, Ar), 126.9 (2×CH, Ar), 126.4 (CH, pyrazole), 122.4 (C, pyrazole), 118.5 (2×CH, Ar), 54.6 (CH_2_), 33.7 (CH), 24.3 (2×CH_3_). Anal. Calcd for C_19_H_20_N_2_O (292.3798): C, 78.92; H, 6.62; N, 9.20. Found: C, 78.63; H, 6.68; N, 9.13.

### (3‐(4‐Isobutylphenyl)‐1‐phenyl‐1*H*‐pyrazol‐4‐yl)methanol (8 f)

Prepared from 1‐phenyl‐3‐(4‐isobutylphenyl)‐1*H*‐pyrazole‐4‐carbaldehyde (**7 f**) (4.00 g, 13.13 mmol). The product was obtained as a greenish brown oil pure enough for use in the following reaction. Yield 3.67 g (91 %). TLC (3 : 1 petroleum ether/EtOAc), R*f*=0.18. ^1^H NMR (DMSO‐d_6_): *δ* 8.32 (s, 1H, pyrazole), 7.82 (d, *J*=7.9 Hz, 2H, Ar), 7.64 (d, *J*=7.8 Hz, 2H, Ar), 7.55 (t, *J*=7.7 Hz, 2H, Ar), 7.43 (t, *J*=7.7 Hz, 1H, para‐Ar), 7.26 (d, *J*=7.9 Hz, 2H, Ar), 5.16 (t, *J*=5.0 Hz, 1H, OH ex), 4.56 (d, *J*=4.9 Hz, 2H, CH_2_), 2.49 (d, *J*=6.8 Hz, 2H, CH_2_), 1.90 (m, 1H, CH), 0.89 (d, *J*=6.8 Hz, 6H, 2×CH_3_). ^13^C NMR (DMSO‐d_6_): *δ* 150.7 (C, pyrazole), 141.5 (C, Ar), 139.9 (C, Ar), 130.9 (C, Ar), 130.0 (2×CH, Ar), 129.6 (2×CH, Ar), 129.0 (CH, para‐Ar), 127.6 (2×CH, Ar), 126.5 (CH, pyrazole), 122.3 (C, pyrazole), 118.5 (2×CH, Ar), 54.6 (CH_2_), 44.8 (CH_2_), 30.1 (CH), 22.6 (2×CH_3_). [ESI‐HRMS] calculated for C_20_H_23_N_2_O: 307.1809 [M+H]^+^. Found: 307.1805 [M+H]^+^.

### (1‐Phenyl‐3‐(4‐tert‐butylphenyl)‐1*H*‐pyrazol‐4‐yl)methanol (8 g)

Prepared from 1‐phenyl‐3‐(4‐(*tert*‐butylphenyl)‐1*H*‐pyrazole‐4‐carbaldehyde (**7 g**) (2.3 g, 7.55 mmol). The product was eluted with petroleum ether – EtOAc 3 : 1 v/v to give the product as a yellow solid, yield 2.0 g (87 %). M.p. 92–94 °C. TLC (1 : 1 petroleum ether/EtOAc), R*f*=0.73. ^1^H NMR (DMSO‐d_6_): δ 8.49 (s, 1H, pyrazole), 7.89 (d, *J*=7.6 Hz, 2H, Ar), 7.82 (d, *J*=8.4 Hz, 2H, Ar), 7.50 (m, 4H, Ar), 7.31 (t, *J*=7.4 Hz, 1H, para‐Ar), 5.18 (t, *J*=4.9 Hz, 1H, OH ex), 4.57 (d, *J*=4.7 Hz, 2H, CH_2_), 1.33 (s, 9H, 3×CH_3_). ^13^C NMR (DMSO‐d_6_): δ 150.8 (C, pyrazole), 140.04 (2×C, Ar), 130.7 (C, Ar), 130.0 (2×CH, Ar), 129.1 (2×CH, Ar), 127.6 (CH, para‐Ar), 126.5 (2×CH, Ar), 125.8 (CH, pyrazole), 122.4 (C, pyrazole), 118.5 (2×CH, Ar), 54.6 (CH_2_), 34.8 (C(CH_3_)_3_), 31.6 (3×CH_3_). [ESI‐HRMS] calculated for C_20_H_23_N_2_O: 307.1805 [M+H]^+^. Found: 307.1797 [M+H]^+^.

### (3‐(Benzo[*d*][1,3]dioxol‐5‐yl)‐1‐phenyl‐1*H*‐pyrazol‐4‐yl)methanol (8 i)

Prepared from 3‐(benzo[*d*][1,3]dioxol‐5‐yl)‐1‐phenyl‐1*H*‐pyrazole‐4‐carbaldehyde (**7 i**) (1.40 g, 4.78 mmol). The product was eluted with petroleum ether – EtOAc 65 : 35 v/v to give the product as a yellow solid, yield 1.06 g (75 %). M.p. 96–98 °C. TLC (1 : 1 petroleum ether/EtOAc), R*f*=0.38. ^1^H NMR (DMSO‐d_6_): δ 8.48 (s, 1H, pyrazole), 7.88 (d, *J*=7.7 Hz, 2H, Ar), 7.51 (t, *J*=7.3 Hz, 2H, Ar), 7.47 (s, 1H, benzo[*d*][1,3]dioxole), 7.41 (d, *J*=1.6 Hz, 1H, benzo[*d*][1,3]dioxole), 7.30 (t, *J*=7.2 Hz, 1H, para‐Ar), 7.02 (d, *J*=7.9 Hz, 1H, benzo[*d*][1,3]dioxole), 6.08 (s, 2H, benzo[*d*][1,3]dioxole), 5.21 (br s, 1H, OH ex), 4.53 (d, 2H, *J*=4.0 Hz, CH_2_). ^13^C NMR (DMSO‐d_6_): δ 150.7 (C, pyrazole), 148.0 (C, benzo[*d*][1,3]dioxole), 147.5 (C, benzo[*d*][1,3]dioxole), 140.0 (C, Ar), 130.0 (2×CH, Ar), 129.2 (CH, para‐Ar), 127.5 (C, benzo[*d*][1,3]dioxole), 126.5 (CH, pyrazole), 121.9 (C, pyrazole), 121.7 (CH, benzo[*d*][1,3]dioxole), 118.8 (2×CH, Ar), 108.9 (CH, benzo[*d*][1,3]dioxole), 108.1 (CH, benzo[*d*][1,3]dioxole), 101.6 (CH_2_, benzo[*d*][1,3]dioxole), 54.5 (CH_2_). [ESI‐HRMS] calculated for C_17_H_15_N_2_O_3_: 295.1077 [M+H]^+^. Found: 295.1077 [M+H]^+^.

### (3‐(1*H*‐Indol‐3‐yl)‐1‐phenyl‐1*H*‐pyrazol‐4‐yl)methanol (8 j)

Prepared from 3‐(1*H*‐indol‐3‐yl)‐1‐phenyl‐1*H*‐pyrazole‐4‐carbaldehyde (**7 j**)^25^ (8.00 g, 27.84 mmol). The product was eluted with petroleum ether – EtOAc 45 : 55 v/v to give the product as a brown waxy solid, yield 5.42 g (67 %). TLC (1 : 2 petroleum ether/EtOAc), R*f*=0.47. ^1^H NMR (DMSO‐d_6_): δ 11.38 (NH, indole), 8.48 (s, 1H, indole), 8.43 (d, *J*=7.2 Hz, 1H, indole), 7.96 (d, *J*=7.6 Hz, 2H, Ar), 7.90 (s, 1H, pyrazole), 7.54 (t, *J*=7.9 Hz, 2H, Ar), 7.49 (d, *J*=7.3 Hz, 1H, indole), 7.28 (t, *J*=7.4 Hz, 1H, para‐Ar), 7.19 (m, 2H, indole), 5.19 (t, *J*=5.1 Hz, 1H, OH ex), 4.64 (d, *J*=4.8 Hz, 2H, CH_2_). ^13^C NMR (DMSO‐d_6_): δ 148.1 (C, Ar), 140.3 (C, indole), 136.8 (C, pyrazole), 130.0 (2×CH, Ar), 127.6 (CH, indole), 126.0 (C, indole), 125.8 (CH, para‐Ar), 122.2 (CH, pyrazole), 122.1 (CH, indole), 121.8 (C, pyrazole), 120.1 (CH, indole), 117.9 (2×CH, Ar), 112.0 (2×CH, indole), 108.6 (C, indole), 55.0 (CH_2_). [ESI‐HRMS] calculated for C_18_H_16_N_3_O: 290.1293 [M+H]^+^. Found: 290.1295 [M+H]^+^.

### General Method for the Preparation of Alcohol Derivatives (19) and (20)

To an ice‐cooled solution of ethyl carboxylate (**17** or **18**)^18^ (5 mmol) in dry THF (15 mL) was added LiAlH_4_ (1 M in THF, 7.5 mmol) dropwise over 25 min. The reaction was then stirred at room temperature overnight then cooled in an ice‐bath and carefully quenched with H_2_O until cessation of effervescence. The reaction mixture was extracted with EtOAc (2×50 mL), then the combined organic layers washed with H_2_O (3×50 mL), dried (MgSO_4_) and evaporated under reduced pressure to give the crude alcohol (**5**) or (**6**) which was was further purified by recrystallisation from CH_2_Cl_2_‐hexane 1 : 1 v/v.

### 1‐(4‐Methoxyphenyl)‐5‐methyl‐1*H*‐pyrazol‐3‐yl)methanol (19)

Prepared from ethyl 1‐(4‐methoxyphenyl)‐5‐methyl‐1*H*‐pyrazole‐3‐carboxylate^18^ (**17**) (1.90 g, 7.18 mmol). Product obtained as a brown solid, yield 1.02 g (64 %). M.p. 115–118 °C. TLC (1 : 1 hexane/EtOAc), R*f*=0.21. ^1^H NMR (DMSO‐d_6_): δ 7.33 (d, *J*=8.9 Hz, 2H, Ar), 6.87 (d, *J*=8.9 Hz, 2H, Ar), 6.06 (s, 1H, pyrazole), 4.99 (t, *J*=5.8 Hz, 1H, ex), 4.39 (d, *J*=5.7 Hz, 2H, CH_2_), 3.78 (s, 3H, OCH_3_), 2.24 (s, 3H, CH_3_). ^13^C‐NMR (DMSO‐d_6_): δ 158.0 (C, Ar), 147.2 (C, pyrazole), 143.6 (C, pyrazole), 133.8 (C, Ar), 123.5 (2×CH, Ar), 114.3 (2×CH, Ar), 106.3 (CH, pyrazole), 55.7 (OCH_3_), 53.0 (CH_2_), 12.1 (CH_3_). Anal. Calcd for C_12_H_14_N_2_O_2_ ⋅ 1H_2_O (220.0563): C, 65.49; H, 6.50; N, 12.73. Found: C, 65.54; H, 6.37; N, 12.83.

### 1‐(4‐Methoxyphenyl)‐3‐methyl‐1*H*‐pyrazol‐5‐yl)methanol (20)

Prepared from ethyl 1‐(4‐methoxyphenyl)‐3‐methyl‐1*H*‐pyrazole‐5‐carboxylate^18^ (**18**) (1.50 g, 5.67 mmol). Product obtained as a brown solid, yield 0.82 g (66 %). M.p. 103–105 °C. TLC (1 : 1 hexane/EtOAc), R*f*=0.43. ^1^H NMR (CDCl_3_): δ 7.39 (d, *J*=9.0 Hz, 2H, Ar), 7.05 (d, *J*=8.9 Hz, 2H, Ar), 6.19 (s, 1H, pyrazole), 5.02 (t, *J*=5.8 Hz, 1H, ex), 4.41 (d, *J*=5.7 Hz, 2H, CH_2_), 3.82 (s, 3H, OCH_3_), 2.26 (s, 3H, CH_3_). ^13^C‐NMR (CDCl_3_): δ 158.6 (C, Ar), 147.8 (C, pyrazole), 144.0 (C, pyrazole), 133.8 (C, Ar), 125.5 (2×CH, Ar), 114.6 (2×CH, Ar), 107.3 (CH, pyrazole), 55.9 (OCH_3_), 54.6 (CH_2_), 12.2 (CH_3_). Anal. Calcd for C_12_H_14_N_2_O_2_ (218.2548): C, 66.04; H, 6.47; N, 12.83. Found: C, 65.80; H, 6.41; N, 12.68.

### General Method for the Preparation of Chlorides (9), (21) and (22)

To an ice‐cooled solution of alcohol (**8** or **19** or **20**) (1 mmol) in dry CH_2_Cl_2_ (5 mL) was added thionyl chloride (10 mmol) dropwise over 25 min. The reaction was stirred at room temperature overnight and then cooled in an ice‐bath and carefully quenched with saturated aqueous NaHCO_3_ in portions until slightly basic (pH 8.0). The organic layer was separated, washed with brine (3×10 mL), H_2_O (2×10 mL), dried (MgSO_4_) and evaporated under reduced pressure to give the crude chloride, which was was purified by petroleum ether – EtOAc gradient column chromatography.

### 4‐(4‐(Chloromethyl)‐1‐phenyl‐1*H*‐pyrazol‐3‐yl)Pyridine (9 a)

Prepared from (1‐phenyl‐3‐(pyridine‐4‐yl)‐1*H*‐pyrazol‐4‐yl)methanol (**8 a**) (0.19 g, 0.75 mmol). The product was obtained as a brown solid that was used immediately in the next step without further purification owing to instability. Yield 0.19 g (75 %). M.p. 78–80 °C. TLC (1 : 4 petroleum ether/EtOAc), R*f*=0.56. ^1^H NMR (DMSO‐d_6_): δ 8.81 (s, 1H, pyrazole), 8.72 (d, *J*=7.3 Hz, 2H, pyridine), 7.91 (d, *J*=7.7 Hz, 2H, Ar), 7.85 (d, *J*=7.2 Hz, 2H, pyridine), 7.56 (d, *J*=7.6 Hz, 2H, Ar), 7.39 (t, *J*=7.4 Hz, 1H, para‐Ar), 5.00 (s, 2H, CH_2_). ^13^C NMR (DMSO‐d_6_): δ 150.5 (2×CH, pyridine), 147.4 (C, Ar), 144.5 (C, Ar), 138.9 (C, Ar), 130.2 (2×CH, Ar), 128.5 (CH, Ar), 124.5 (CH, Ar), 122.4 (2×CH, Ar), 119.6 (2×CH, Ar), 116.2 (C, Ar), 37.9 (CH_2_).

### 3‐(4‐(Chloromethyl)‐1‐phenyl‐1*H*‐pyrazol‐3‐yl)Pyridine (9 b)

Prepared from (1‐phenyl‐3‐(pyridine‐3‐yl)‐1*H*‐pyrazol‐4‐yl)methanol (**8 b**) (0.87 g, 3.49 mmol). The product was obtained as a pale orange solid that was used immediately in the next step without further purification owing to instability. Yield 0.8 g (85 %). M.p. 52 °C (decomp.). TLC (1 : 4 petroleum ether/EtOAc), R*f*=0.76. ^1^H NMR (DMSO‐d_6_): δ 9.14 (s, 1H, pyridine), 8.75 (d, *J*=6.5 Hz, 1H, pyridine), 8.67 (s, 1H, pyrazole), 8.52 (d, *J*=8.1 Hz, 1H, pyridine), 8.01 (d, *J*=7.7 Hz, 2H, Ar), 7.72 (m, 3H, 1×pyridine, 2×Ar), 7.40 (t, *J*=7.4 Hz, 1H, para‐Ar), 4.98 (s, 2H, CH_2_). ^13^C NMR (DMSO‐d_6_): δ 149.1 (CH, pyridine), 148.4 (CH, pyridine), 148.0 (C, Ar), 139.5 (C, pyrazole), 135.4 (CH, pyridine), 129.0 (2×CH, Ar), 128.2 (C, pyrazole), 128.2 (CH, pyridine), 126.9 (2×CH, Ar), 124.1 (CH, para‐Ar), 119.2 (CH, pyrazole), 116.8 (C, pyridine), 37.9 (CH_2_).

### 4‐(Chloromethyl)‐3‐(4‐ethylphenyl)‐1‐phenyl‐1*H*‐pyrazole (9 c)

Prepared from (3‐(4‐ethylphenyl)‐1‐phenyl‐1*H*‐pyrazol‐4‐yl)methanol (**8 c**) (0.80 g, 2.87 mmol). The product was eluted with petroleum ether – EtOAc 9 : 1 v/v to give the product as a yellow solid, yield 0.51 g (82 %). M.p. 96–98 °C. TLC (2 : 1 petroleum ether/EtOAc), R*f*=0.94. ^1^H NMR (DMSO‐d_6_): δ 8.74 (s, 1H, pyrazole), 7.89 (d, *J*=7.7 Hz, 2H, Ar), 7.77 (d, *J*=7.8 Hz, 2H, Ar), 7.54 (t, *J*=7.8 Hz, 1H, para‐Ar), 7.36 (d, *J*=8.5 Hz, 4H, Ar), 4.91 (s, 1H, CH_2_), 2.68 (q, *J*=7.4 Hz, 2H, CH_2_), 1.23 (t, *J*=7.4 Hz, 3H, CH_3_). ^13^C NMR (DMSO‐d_6_): δ 151.0 (C, pyrazole), 144.5 (C, Ar), 139.6 (C, Ar), 130.7 (C, pyrazole), 130.1 (C, Ar), 130.1 (2×CH, Ar), 128.7 (CH, para‐Ar,), 127.8 (2×CH, Ar), 127.1 (2×CH, Ar), 118.8 (CH, pyrazole), 117.9 (2×CH, Ar), 38.3 (CH_2_), 28.43 (CH_2_), 16.0 (CH_3_). [ESI‐HRMS] calculated for C_18_H_18_ClN_2_: 297.6373 [M+H]^+^. Found: 297.6362 [M+H]^+^.

### 4‐(Chloromethyl)‐1‐phenyl‐3‐(4‐propylphenyl)‐1*H*‐pyrazole (9 d)

Prepared from (1‐phenyl‐3‐(4‐propylphenyl)‐1*H*‐pyrazol‐4‐yl)methanol (**8 d**) (0.70 g, 2.39 mmol). The product was eluted with petroleum ether – EtOAc 85 : 15 v/v to give the product as a brown solid, yield 0.33 g (82 %). M.p. 82–84 °C. TLC (2 : 1 petroleum ether/EtOAc), R*f*=0.88. ^1^H NMR (DMSO‐d_6_): δ 8.09 (s, 1H, pyrazole), 7.77 (m, 4H, Ar), 7.49 (t, *J*=8.1 Hz, 1H, para‐Ar), 7.33 (m, 4H, Ar), 4.76 (s, 2H, CH_2_), 2.67 (t, *J*=7.4 Hz, 2H, CH_2_), 1.72 (sext, *J*=7.2 Hz, 2H, CH_2_), 1.00 (t, *J*=7.3 Hz, 3H, CH_3_). ^13^C NMR (DMSO‐d_6_): δ 151.8 (C, pyrazole), 143.1 (C, Ar), 139.8 (C, Ar), 129.8 (C, Ar), 129.5 (2×CH, Ar), 128.9 (2×CH, Ar), 128.5 (CH, para‐Ar), 127.9 (2×CH, Ar), 126.7 (CH, pyrazole), 119.1 (2×CH, Ar), 117.7 (C, pyrazole), 37.9 (CH_2_), 37.3 (CH_2_), 24.5 (CH_2_), 13.8 (CH_3_). [ESI‐HRMS] calculated for C_19_H_20_ClN_2_: 311.1312 [M+H]^+^. Found: 311.1310 [M+H]^+^.

### 4‐(Chloromethyl)‐3‐(4‐isopropylphenyl)‐1‐phenyl‐1*H*‐pyrazole (9 e)

Prepared from (1‐phenyl‐3‐(4‐isopropylphenyl)‐1*H*‐pyrazol‐4‐yl)methanol (**8 e**) (0.62 g, 2.11 mmol). The product was eluted with petroleum ether – EtOAc 85 : 15 v/v to give the product as a brown solid, yield 0.53 g (80 %). M.p. 114–116 °C. TLC (2 : 1 petroleum ether/EtOAc), R*f*=0.85. ^1^H NMR (DMSO‐d_6_): δ 8.74 (s, 1H, pyrazole), 7.89 (d, *J*=7.8 Hz, 2H, Ar), 7.78 (d, *J*=8.1 Hz, 2H, Ar), 7.53 (t, *J*=7.7 Hz, 2H, Ar), 7.39 (d, *J*=8.1 Hz, 2H, Ar), 7.34 (t, *J*=7.3 Hz, 1H, para‐Ar), 4.90 (s, 2H, CH_2_), 2.95 (sept, *J*=6.9 Hz, 1H, CH), 1.25 (d, *J*=6.9 Hz, 6H, 2×CH_3_). ^13^C NMR (DMSO‐d_6_): δ 151.0 (C, pyrazole), 149.1 (C, Ar), 139.7 (C, Ar), 130.7 (CH, para‐Ar), 130.3 (C, Ar), 130.1 (2×CH, Ar), 127.9 (2×CH, Ar), 127.2 (2×CH, Ar), 127.1 (CH, pyrazole), 118.8 (2×CH, Ar), 117.9 (C, pyrazole), 38.3 (CH_2_), 33.7 (CH), 24.3 (2×CH_3_). [ESI‐HRMS] calculated for C_19_H_20_ClN_2_: 311.1312 [M+H]^+^. Found: 311.1313 [M+H]^+^.

### 4‐(Chloromethyl)‐3‐(4‐isobutylphenyl)‐1‐phenyl‐1*H*‐pyrazole (9 f)

Prepared from (1‐phenyl‐3‐(4‐isobutylphenyl)‐1*H*‐pyrazol‐4‐yl)methanol (**8 f**) (2.20 g, 7.17 mmol). The product was obtained as a greenish brown solid pure enough for use in the next reaction. Yield 1.90 g (82 %). M.p. 100–102 °C. TLC (3 : 1 petroleum ether/EtOAc), R*f*=0.62. ^1^H NMR (DMSO‐d_6_): δ 8.46 (s, 1H, pyrazole), 7.88 (d, *J*=7.9 Hz, 2H, Ar), 7.79 (d, *J*=7.7 Hz, 2H, Ar), 7.51 (t, *J*=7.5 Hz, 2H, Ar), 7.30 (t, *J*=7.5 Hz, 1H, para‐Ar), 7.25 (d, *J*=7.8 Hz, 2H, Ar), 4.95 (s, 2H, CH_2_), 2.49 (d, *J*=7.3 Hz, 2H, CH_2_), 1.87 (m, 1H, CH), 0.89 (d, *J*=6.5 Hz, 6H, 2×CH_3_). ^13^C NMR (DMSO‐d_6_): δ 149.9 (C, pyrazole), 142.2 (C, Ar), 139.9 (C, Ar), 131.5 (2×CH, Ar), 129.9 (C, Ar), 129.5 (2×CH, Ar), 126.5 (CH, para‐Ar), 124.8 (2×CH, Ar), 123.0 (CH, pyrazole), 120.3 (2×CH, Ar), 119.2 (C, pyrazole), 38.3 (CH_2_), 36.0 (CH_2_), 29.0 (CH), 22.7 (2×CH_3_). [ESI‐HRMS] calculated for C_20_H_22_ClN_2_: 325.1472 [M+H]^+^. Found: 325.1478 [M+H]^+^.

### 4‐(Chloromethyl)‐3‐(4‐tert‐butylphenyl)‐1‐phenyl‐1*H*‐Pyrazole (9 g)

Prepared from (1‐phenyl‐3‐(4‐*tert*‐butylphenyl)‐1*H*‐pyrazol‐4‐yl)methanol (**8 g**) (2.00 g, 6.52 mmol). The product was obtained as a pale yellow solid pure enough for use in the next reaction. Yield 1.46 g (69 %). M.p. 132–134 °C. TLC (1 : 1 petroleum ether/EtOAc), R*f*=0.94. ^1^H NMR (DMSO‐d_6_) δ 8.09 (s, 1H, pyrazole), 7.89 (d. *J*=7.6 Hz, 2H, Ar), 7.79 (d, *J*=8.4 Hz, 2H, Ar), 7.53 (m, 4H, Ar), 7.35 (t, *J*=6.5 Hz, 1H, para‐Ar), 4.91 (s, 2H, CH_2_), 1.33 (s, 9H, 3×CH_3_). ^13^C NMR (DMSO‐d_6_): δ 151.3 (C, Ar), 150.9 (C, pyrazole), 139.7 (C, Ar), 130.7 (CH, para‐Ar), 130.1 (2×CH, Ar), 129.9 (C, Ar), 127.6 (2×CH, Ar), 127.0 (CH, pyrazole), 126.0 (2×CH, Ar), 118.8 (2×CH, Ar), 118.0 (C, pyrazole), 38.3 (CH_2_), 34.9 (C(CH_3_)_3_), 31.5 (3×CH_3_). [ESI‐HRMS] calculated for C_20_H_22_ClN_2_: 325.1466 [M+H]^+^. Found: 325.1461 [M+H]^+^.

### 3‐(Benzo[d][1,3]dioxol‐5‐yl)‐4‐(chloromethyl)‐1‐phenyl‐1*H*‐pyrazole (9 i)

Prepared from (3‐(benzo[*d*][1,3]dioxol‐5‐yl)‐1‐phenyl‐1*H*‐pyrazol‐4‐yl)methanol (**8 i**) (0.90 g, 3.068 mmol). The product was eluted with petroleum ether – EtOAc 85 : 15 v/v to give the product as a yellow solid, yield 0.75 g (76 %). M.p. 158–159 °C. TLC (1 : 1 petroleum ether/EtOAc), R*f*=0.81. ^1^H NMR (DMSO‐d_6_): δ 8.54 (s, 1H, pyrazole), 7.81 (d, *J*=8.0 Hz, 2H, Ar), 7.54 (t, *J*=7.8 Hz, 2H, Ar), 7.42 (t, *J*=7.4 Hz, 1H, Ar), 7.37 (s, 1H, benzo[*d*][1,3]dioxole), 7.28 (d, *J*=1.6 Hz, 1H, benzo[*d*][1,3]dioxole), 6.96 (d, *J*=8.1 Hz, 1H, benzo[*d*][1,3]dioxole), 6.07 (s, 2H, benzo[*d*][1,3]dioxole), 4.92 (s, 2H, CH_2_). ^13^C NMR (DMSO‐d_6_): δ 151.4 (C, pyrazole), 148.0 (C, Ar), 147.9 (C, benzo[*d*][1,3]dioxole), 139.7 (C, benzo[*d*][1,3]dioxole), 129.5 (2×CH, Ar), 128.6 (CH, para‐Ar), 126.7 (CH, pyrazole), 126.4 (C, benzo[*d*][1,3]dioxole), 121.9 (CH, benzo[*d*][1,3]dioxole), 119.1 (2×CH, Ar), 117.5 (C, pyrazole), 108.6 (CH, benzo[*d*][1,3]dioxole), 108.5 (CH, benzo[*d*][1,3]dioxole), 101.2 (CH_2_, benzo[*d*][1,3]dioxole), 37.2 (CH_2_). [ESI‐HRMS] calculated for C_17_H_14_ClN_2_O_2_: 313.0931 [M+H]^+^. Found: 313.0919 [M+H]^+^.

### 2‐Chloro‐3‐(4‐(chloromethyl)‐1‐phenyl‐1*H*‐pyrazol‐3‐yl)‐1*H*‐indole (9 j)

Prepared from (3‐(1*H*‐indol‐3‐yl)‐1‐phenyl‐1*H*‐pyrazol‐4‐yl)methanol (**8 j**) (1.50 g, 5.18 mmol). The product was eluted with petroleum ether – EtOAc 85 : 15 v/v to give the product as a brown waxy solid, yield 0.90 g (51 %). TLC (1 : 1 petroleum ether/EtOAc), R*f*=0.91. ^1^H NMR (CDCl_3_): δ 11.07 (NH, indole), 8.27 (s, 1H, pyrazole), 7.88 (d, *J*=7.6 Hz, 2H, Ar), 7.59 (t, *J*=7.9 Hz, 2H, Ar), 7.50 (d, *J*=7.3 Hz, 1H, indole), 7.43 (t, *J*=7.5 Hz, 1H, indole), 7.34 (d, *J*=8.6 Hz, 1H, indole), 7.25 (t, *J*=7.3 Hz, 1H, indole), 7.16 (t, *J*=7.5 Hz, 1H, para‐Ar), 4.69 (s, 2H, CH_2_). ^13^C NMR (CDCl_3_): δ 145.8 (C, Ar), 139.8 (C, indole), 136.7 (C, pyrazole), 134.5 (C, indole), 129.8 (2×CH, Ar), 129.5 (CH, para‐Ar), 127.5 (CH, indole), 127.4 (C, pyrazole), 127.3 (C, indole), 123.6 (CH, pyrazole), 120.8 (CH, indole), 120.7 (C, indole), 119.9 (2×CH, Ar), 119.5 (CH, indole), 111.7 (CH, indole), 36.8 (CH_2_). [ESI‐HRMS] calculated for C_18_H_14_Cl_2_N_3_: 342.0565 [M+H]^+^. Found: 242.0550 [M+H]^+^.

### 3‐(Chloromethyl)‐1‐(4‐methoxyphenyl)‐5‐methyl‐1*H*‐pyrazole (21)

Prepared from 1‐(4‐methoxyphenyl)‐5‐methyl‐1*H*‐pyrazol‐3‐yl)methanol (**19**) (2.0 g, 9.16 mmol). The product was eluted with petroleum ether – EtOAc 40 : 60 v/v to give the product as a brown oil, yield 1.36 g (63 %). TLC (1 : 1 hexane/EtOAc), R*f*=0.85. ^1^H NMR (DMSO‐d_6_): δ δ 7.52 (d, *J*=9.0 Hz, 2H, Ar), 7.13 (d, *J*=9.0 Hz, 2H, Ar), 6.44 (s, 1H, pyrazole), 4.87 (s, 2H, CH_2_), 3.89 (s, 3H, OCH_3_), 2.29 (s, 3H, CH_3_). ^13^C‐NMR (DMSO‐d_6_): δ 159.0 (C, Ar), 148.5 (C, pyrazole), 140.6 (C, pyrazole), 132.7 (C, Ar), 126.6 (2×CH, Ar), 114.7 (2×CH, Ar), 106.7 (CH, pyrazole), 55.9 (OCH_3_), 39.5 (CH_2_), 12.4 (CH_3_). [ESI‐HRMS] calculated for C_12_H_14_ClN_2_O: 237.0795 [M+H]^+^. Found: 237.0799 [M+H]^+^.

### 5‐(Chloromethyl)‐1‐(4‐methoxyphenyl)‐3‐methyl‐1*H*‐pyrazole (22)

Prepared from 1‐(4‐methoxyphenyl)‐3‐methyl‐1*H*‐pyrazol‐5‐yl)methanol (**20**) (2.0 g, 9.16 mmol). The product was eluted with petroleum ether – EtOAc 40 : 60 v/v to give the product as a yellow solid, yield 1.43 g (66 %). M.p. 48–50 °C. TLC (1 : 1 hexane/EtOAc), R*f*=0.71. ^1^H NMR (DMSO‐d_6_): 7.49 (d, *J*=9.0 Hz, 2H, Ar), 7.08 (d, *J*=9.0 Hz, 2H, Ar), 6.39 (s, 1H, pyrazole), 4.76 (s, 2H, CH_2_), 3.83 (s, 3H, OCH_3_), 2.22 (s, 3H, CH_3_). ^13^C‐NMR (DMSO‐d_6_): δ 159.5 (C, Ar), 148.7 (C, pyrazole), 140.9 (C, pyrazole), 133.0 (C, Ar), 126.8 (2×CH, Ar), 115.0 (2×CH, Ar), 106.9 (CH, pyrazole), 56.2 (OCH_3_), 39.8 (CH_2_), 12.7 (CH_3_). [ESI‐HRMS] calculated for C_12_H_14_ClN_2_O: 237.0795 [M+H]^+^. Found: 237.0799 [M+H]^+^.

### General Method for the Preparation of Imidazole Derivatives (10), (23) and (25) and Triazole Derivatives (11), (24) and (26)

To a stirred suspension of K_2_CO_3_ (4 mmol) in dry acetonitrile (10 mL) was added imidazole (4 mmol) or triazole (4 mmol). The reaction mixture was refluxed at 45 °C for 1 h. After cooling to room temperature the chloromethyl pyrazole compound (**9**, **21** or **22**) (1 mmol) was added and the reaction mixture refluxed at 70 °C overnight. The solvent was evaporated under reduced pressure and the resulting mixture diluted with EtOAc (50 mL) and washed with brine (3×20 mL) and H_2_O (3×20 mL). The organic layer was dried (MgSO_4_) and evaporated under reduced pressure to give the crude imidazole or triazole, which was purified by gradient column chromatography.

### 4‐(4‐((1*H*‐Imidazol‐1‐yl)methyl)‐1‐phenyl‐1*H*‐pyrazol‐3‐yl)pyridine (10 a)

Prepared from 4‐(4‐(chloromethyl)‐1‐phenyl‐1*H*‐pyrazol‐3‐yl)pyridine (**9 a**) (0.20 g, 0.74 mmol). The product was eluted with CH_2_Cl_2_−MeOH−Et_3_N 91.5 : 7.5 : 1 v/v/v to give the product as a yellow solid, yield 0.15 g (67 %). M.p. 80–82 °C. TLC (9 : 1 CH_2_Cl_2_/MeOH), R*f*=0.42. ^1^H NMR (DMSO‐d_6_): δ 8.65 (d, *J*=5.7 Hz, 2H, pyridine), 8.59 (s, 1H, pyrazole), 7.90 (d, *J*=7.0 Hz, 2H, Ar), 7.71 (s, 1H, imidazole), 7.66 (d, *J*=5.3 Hz, 2H, pyridine), 7.55 (t, *J*=7.4 Hz, 2H, Ar), 7.38 (t, *J*=7.2 Hz, 1H, para‐Ar), 7.16 (s, 1H, imidazole), 6.89 (s, 1H, imidazole), 5.40 (s, 2H, CH_2_). ^13^C NMR (DMSO‐d_6_): δ 150.6 (2×CH, pyridine), 147.9 (C, pyridine), 140.1 (C, Ar), 139.5 (C, pyrazole), 135.6 (CH, imidazole), 130.7 (CH, imidazole), 130.1 (2×CH, Ar), 129.2 (CH, pyrazole), 127.5 (CH, para‐Ar), 122.1 (2×CH, pyridine), 119.7 (CH, imidazole), 119.1 (2×CH, Ar), 118.4 (C, pyrazole), 40.8 (CH_2_). [ESI‐HRMS] calculated for C_18_H_16_N_5_: 302.1406 [M+H]^+^. Found: 302.1392 [M+H]^+^.

### 3‐(4‐((1*H*‐Imidazol‐1‐yl)methyl)‐1‐phenyl‐1*H*‐pyrazol‐3‐yl)pyridine (10 b)

Prepared from 3‐(4‐(chloromethyl)‐1‐phenyl‐1*H*‐pyrazol‐3‐yl)pyridine (**9 b**) (0.50 g, 1.85 mmol). The product was eluted with CH_2_Cl_2_−MeOH−Et_3_N 89 : 10 : 1 v/v/v to give the product as a yellow oil, yield 0.30 g (54 %). TLC (9 : 1 CH_2_Cl_2_/MeOH), R*f*=0.46. ^1^H NMR (CDCl_3_): δ 8.86 (s, 1H, pyridine), 8.65 (d, *J*=4.9 Hz, 1H, pyridine), 7.89 (d, *J*=7.9 Hz, 1H, pyridine), 7.86 (s, 1H, pyrazole), 7.65 (d, *J*=7.6 Hz, 2H, Ar), 7.69 (s, 1H, imidazole), 7.56 (s, 1H, imidazole), 7.49 (t, *J*=8.4 Hz, 2H, Ar), 7.39 (m, 1H, pyridine), 7.35 (t, *J*=7.4 Hz, 1H, para‐Ar), 7.97 (s, 1H, imidazole), 5.25 (s, 2H, CH_2_). ^13^C NMR (CDCl_3_): δ 149.6 (CH, pyridine), 148.6 (CH, pyridine), 148.2 (C, Ar), 139.4 (C, pyrazole), 135.1 (CH, pyridine), 129.9 (CH, imidazole), 129.6 (2×CH, Ar), 128.5 (C, pyrazole), 128.0 (CH, pyridine), 127.3 (2×CH, Ar), 123.7 (CH, para‐Ar), 123.7 (CH, imidazole), 119.2 (CH, pyrazole), 119.1 (CH, imidazole), 116.7 (C, pyridine), 41.7 (CH_2_). [ESI‐HRMS] calculated for C_18_H_16_N_5_: 302.1400 [M+H]^+^. Found: 302.1399 [M+H]^+^.

### 4‐((1*H*‐Imidazol‐1‐yl)methyl)‐3‐(4‐ethylphenyl)‐1‐phenyl‐1*H*‐pyrazole (10 c)

Prepared from 4‐(chloromethyl)‐3‐(4‐ethylphenyl)‐1‐phenyl‐1*H*‐pyrazole (**9 c**) (0.50 g, 1.68 mmol). The product was eluted with CH_2_Cl_2_−MeOH 95 : 5 v/v to give the product as a yellow solid, yield 0.32 g (58 %). M.p. 74–76 °C. TLC (9 : 1 CH_2_Cl_2_/MeOH), R*f*=0.83. ^1^H NMR (DMSO‐d_6_): δ 7.81 (s, 1H, pyrazole), 7.73 (d, *J*=9 Hz, 2H, Ar), 7.65 (s, 1H, imidazole), 7.52 (d, *J*=9 Hz, 2H, Ar), 7.47 (t, *J*=8.3 Hz, 1H, para‐Ar), 7.32 (m, 4H, Ar), 7.12 (s, 1H, imidazole), 6.97 (s, 1H, imidazole), 5.24 (s, 2H, CH_2_), 2.72 (q, *J*=7.6 Hz, 2H, CH_2_), 1.29 (t, *J*=7.5 Hz, 3H, CH_3_). ^13^C NMR (DMSO‐d_6_): δ 151.5 (C, pyrazole), 144.8 (C, Ar), 139.7 (C, Ar), 129.7 (C, pyrazole), 129.7 (CH, imidazole), 129.5 (C, Ar), 128.4 (2×CH, Ar), 127.8 (CH, para‐Ar), 127.6 (2×CH, Ar), 127.4 (2×CH, Ar), 126.7 (CH, imidazole), 119.0 (CH, imidazole), 117.7 (CH, pyrazole), 116.3 (2×CH, Ar), 41.8 (CH_2_), 28.7 (CH_2_), 15.5 (CH_3_). Anal. Calcd for C_21_H_20_N_4_ (328.4158): C, 76.80; H, 6.14; N, 17.05. Found: C, 76.68; H, 6.02; N, 17.18.

### 4‐((1*H*‐Imidazol‐1‐yl)methyl)‐1‐phenyl‐3‐(4‐propylphenyl)‐1*H*‐pyrazole (10 d)

Prepared from 4‐(chloromethyl)‐3‐(4‐propylphenyl)‐1‐phenyl‐1*H*‐pyrazole (**9 d**) (0.50 g, 1.61 mmol). The product was eluted with CH_2_Cl_2_−MeOH 95 : 5 v/v to give the product as a brown oil, yield 0.36 g (65 %). TLC (9 : 1 CH_2_Cl_2_/MeOH), R*f*=0.87. ^1^H NMR (CDCl_3_): δ 9.03 (s, 1H, pyrazole), 8.37 (s, 1H, imidazole), 7.78 (d, *J*=8.2 Hz, 2H, Ar), 7.48 (m, 4H, Ar), 7.35 (t, *J*=7.2 Hz, 1H, para‐Ar), 7.30 (d, *J*=8.0 Hz, 2H, Ar), 7.28 (s, 1H, imidazole), 6.93 (s, 1H, imidazole), 5.54 (s, 2H, CH_2_), 2.67 ( t, *J*=7.7 Hz, 2H, CH_2_), 1.70 (sext, *J*=7.4 Hz, 2H, CH_2_), 0.98 (t, *J*=7.3 Hz, 3H, CH_3_). ^13^C NMR (CDCl_3_): *δ* 151.5 (C, pyrazole), 143.2 (C, Ar), 139.7 (C, Ar), 137.0 (CH, imidazole), 129.7 (C, Ar), 129.6 (CH, imidazole), 129.5 (2×CH, Ar), 129.0 (2×CH, Ar), 127.7 (CH, imidazole), 127.6 (2×CH, Ar), 126.7 (CH, para‐Ar), 125.2 (CH, pyrazole), 117.0 (2×CH, Ar), 116.3 (C, pyrazole), 41.8 (CH_2_), 37.8 (CH_2_), 24.5 (CH_2_), 13.8 (CH_3_). [ESI‐HRMS] calculated for C_22_H_23_N_4_: 343.1917 [M+H]^+^. Found: 343.1915 [M+H]^+^.

### 4‐((1*H*‐Imidazol‐1‐yl)methyl)‐3‐(4‐isopropylphenyl)‐1‐phenyl‐1*H*‐pyrazole (10 e)

Prepared from 4‐(chloromethyl)‐3‐(4‐isopropylphenyl)‐1‐phenyl‐1*H*‐pyrazole (**9 e**) (0.50 g, 1.61 mmol). The product was eluted with CH_2_Cl_2_−MeOH 97.5 : 2.5 v/v to give the product as a brown oil, yield 0.29 g (52 %). TLC (9 : 1 CH_2_Cl_2_/MeOH), R*f*=0.74. ^1^H NMR (DMSO‐d_6_): δ 8.51 (s, 1H, pyrazole), 7.87 (d, *J*=8.1 Hz, 2H, Ar), 7.67 (s, 1H, imidazole), 7.56 (d, *J*=8.1 Hz, 2H, Ar), 7.53 (t, *J*=7.6 Hz, 1H, para‐Ar), 7.35 (d, *J*=8.1 Hz, 4H, Ar), 7.15 (s, 1H, imidazole), 6.90 (s, 1H, imidazole), 5.30 (s, 2H, CH_2_), 2.95 (sept, *J*=6.9 Hz, 1H, CH), 1.25 (d, *J*=6.9 Hz, 6H, 2×CH_3_). ^13^C NMR (DMSO‐d_6_): δ 150.8 (C, pyrazole), 149.0 (C, Ar), 139.8 (C, Ar), 130.4 (C, Ar), 130.1 (CH, imidazole), 130.0 (2×CH, Ar), 129.1 (CH, imidazole), 127.9 (2×CH, Ar), 127.1 (2×CH, Ar), 126.9 (CH, para‐Ar), 123.1 (CH, pyrazole), 119.7 (CH, imidazole), 118.8 (2×CH, Ar), 117.2 (C, pyrazole), 41.0 (CH_2_), 33.7 (CH), 24.3 (2×CH_3_). [ESI‐HRMS] calculated for C_22_H_23_N_4_: 343.1917 [M+H]^+^. Found: 343.1917 [M+H]^+^.

### 4‐((1*H*‐Imidazol‐1‐yl)methyl)‐3‐(4‐isobutylphenyl)‐1‐phenyl‐1*H*‐pyrazole (10 f)

Prepared from 3‐(4‐isobutylphenyl)‐4‐(chloromethyl)‐1‐phenyl‐1*H*‐pyrazole (**9 f**) (0.85 g, 2.61 mmol). The product was eluted with CH_2_Cl_2_−MeOH 97.5 : 2.5 v/v to give the product as a yellow oil, yield 0.58 g (62 %). TLC (95 : 5 CH_2_Cl_2_/MeOH), R*f*=0.51. ^1^H NMR (CDCl_3_): δ 7.77 (s, 1H, pyrazole), 7.70 (d, *J*=8.2 Hz, 2H, Ar), 7.54 (s, 1H, imidazole), 7.48 (d, *J*=7.8 Hz, 2H, Ar), 7.42 (t, *J*=7.8 Hz, 2H, Ar), 7.27 (t, *J*=7.5 Hz, 1H, para‐Ar), 7.22 (d, *J*=8.1 Hz, 2H, Ar), 7.07 (s, 1H, imidazole), 6.91 (s, 1H, imidazole), 5.16 (s, 2H, CH_2_), 2.52 (d, *J*=7.1 Hz, 2H, CH_2_), 1.90 (m, 1H, CH), 0.93 (d, *J*=6.6 Hz, 6H, 2×CH_3_)_._^13^C NMR (CDCl_3_): δ 151.5 (C, pyrazole), 142.3 (C, Ar), 139.6 (C, Ar), 136.9 (CH, imidazole), 129.7 (C, Ar), 129.6 (2×CH, Ar), 129.5 (CH, imidazole), 129.5 (2×CH, Ar), 127.6 (CH, imidazole), 127.6 (2×CH, Ar), 126.9 (CH, pyrazole), 126.7 (CH, para‐Ar), 118.9 (2×CH, Ar), 116.2 (C, pyrazole), 45.2 (CH_2_), 41.7 (CH_2_), 30.2 (CH), 22.4 (2×CH_3_). [ESI‐HRMS] calculated for C_23_H_25_N_4_: 357.2079 [M+H]^+^. Found: 357.2086 [M+H]^+^.

### 4‐((1*H*‐Imidazol‐1‐yl)methyl)‐3‐(4‐(tert‐butyl)phenyl)‐1‐phenyl‐1*H*‐pyrazole (10 g)

Prepared from 3‐(4‐(*tert*‐butyl)phenyl)‐4‐(chloromethyl)‐1‐phenyl‐1*H*‐pyrazole (**9 g**) (0.50 g, 1.53 mmol). The product was eluted with CH_2_Cl_2_−MeOH 97.5 : 2.5 v/v to give the product as a yellow solid, yield 0.31 g (57 %). M.p. 116–117 °C. TLC (95 : 5 CH_2_Cl_2_/MeOH), R*f*=0.72. ^1^H NMR (DMSO‐d_6_): δ 8.52 (s, 1H, pyrazole), 7.87 (d, *J*=7.8 Hz, 2H, Ar), 7.68 (s, 1H, imidazole), 7.57 (d, *J*=8.3 Hz, 2H, Ar), 7.53 (t, *J*=7.7 Hz, 2H, Ar), 7.49 (d, *J*=8.3 Hz, 2H, Ar), 7.34 (t, *J*=7.3 Hz, 1H, para‐Ar), 7.16 (s, 1H, imidazole), 6.91 (s, 1H, imidazole), 5.30 (s, 2H, CH_2_), 1.32 (s, 9H, 3×CH_3_). ^13^C NMR (DMSO‐d_6_): δ 151.2 (C, Ar), 150.7 (C, pyrazole), 139.8 (C, Ar), 139.8 (C, Ar), 137.5 (CH, imidazole), 130.1 (2×CH, Ar), 130.0 (CH, imidazole), 129.1 (CH, para‐Ar), 127.6 (2×CH, Ar), 126.9 (CH, pyrazole), 126.0 (2×CH, Ar), 119.7 (CH, imidazole), 118.8 (2×CH, Ar), 117.2 (C, pyrazole), 41.0 (CH_2_), 34.9 (C(CH_3_)_3_), 31.5 (3×CH_3_). Anal. Calcd for C_23_H_24_N_4_ (356.4694): C, 77.50; H, 6.79; N, 15.71. Found: C, 77.92; H, 7.04; N, 15.88.

### 4‐((1*H*‐Imidazol‐1‐yl)methyl)‐3‐([1,1′‐biphenyl]‐4‐yl)‐1‐phenyl‐1*H*‐pyrazole (10 h)

Prepared from 3‐([1,1′‐biphenyl]‐4‐yl)‐4‐(chloromethyl)‐1‐phenyl‐1*H*‐pyrazole (**9 h**)^26^ (0.40 g, 1.16 mmol). The product was eluted with CH_2_Cl_2_−MeOH 98 : 2 v/v to give the product as a yellow solid, yield 0.20 g (46 %). M.p. 114–115 °C. TLC (95 : 5 CH_2_Cl_2_/MeOH), R*f*=0.65. ^1^H NMR (DMSO‐d_6_): δ 8.56 (s, 1H, pyrazole), 7.90 (d, *J*=7.6 Hz, 2H, Ar), 7.77 (m, 6H, Ar), 7.71 (s, 1H, imidazole), 7.54 (t, *J*=7.9 Hz, 2H, Ar), 7.50 (t, *J*=7.8 Hz, 2H, Ar), 7.40 (t, *J*=7.8 Hz, 1H, para‐Ar), 7.36 (t, *J*=7.7 Hz, 1H, para‐Ar), 7.18 (s, 1H, imidazole), 6.91 (s, 1H, imidazole), 5.37 (s, 2H, CH_2_). ^13^C NMR (DMSO‐d_6_): δ 150.3 (C, pyrazole), 140.3 (C, Ar), 140.0 (C, Ar), 139.7 (C, Ar), 132.0 (2×CH, Ar), 130.2 (2×CH, Ar), 130.1 (CH, imidazole), 129.5 (2×CH, Ar), 129.1 (CH, imidazole), 128.4 (2×CH, Ar), 128.2 (2×CH, Ar), 127.4 (2×CH, Ar), 127.1 (2×CH, Ar), 127.0 (CH, para‐Ar), 119.7 (CH, imidazole), 118.9 (CH, para‐Ar), 117.5 (C, pyrazole), 41.0 (CH_2_). [ESI‐HRMS] calculated for C_25_H_21_N_4_: 377.1766 [M+H]^+^. Found: 377.1770 [M+H]^+^.

### 4‐((1*H*‐Imidazol‐1‐yl)methyl)‐3‐(benzo[*d*][1,3]dioxol‐5‐yl)‐1‐phenyl‐1*H*‐pyrazole (10 i)

Prepared from 3‐(benzo[*d*][1,3]dioxol‐5‐yl)‐4‐(chloromethyl)‐1‐phenyl‐1*H*‐pyrazole (**9 i**) (0.60 g, 1.92 mmol). The product was eluted with CH_2_Cl_2_−MeOH 98 : 2 v/v to give the product as a yellow solid, yield 0.32 g (49 %). M.p. 62–64 °C. TLC (95 : 5 CH_2_Cl_2_/MeOH), R*f*=0.47. ^1^H NMR (CDCl_3_): δ 7.72 (s, 1H, pyrazole), 7.65 (d, *J*=7.9 Hz, 2H, Ar), 7.50 (s, 1H, imidazole), 7.39 (t, *J*=7.6 Hz, 2H, Ar), 7.24 (t, *J*=7.4 Hz, 1H, para‐Ar), 7.09 (s, 1H, imidazole), 7.06 (s, 1H, benzo[*d*][1,3]dioxole), 6.94 (d, *J*=1.6 Hz, 1H, benzo[*d*][1,3]dioxole), 6.89 (s, 1H, imidazole), 6.82 (d, *J*=8.0 Hz, 1H, benzo[*d*][1,3]dioxole), 5.93 (s, 2H, benzo[*d*][1,3]dioxole), 5.10 (s, 2H, CH_2_). ^13^C NMR (CDCl_3_): δ 151.0 (C, pyrazole), 148.1 (C, benzo[*d*][1,3]dioxole), 147.9 (C, benzo[*d*][1,3]dioxole), 139.5 (C, Ar), 137.0 (CH, imidazole), 129.7 (CH, imidazole), 129.5 (2×CH, Ar), 127.6 (CH, para‐Ar), 126.7 (CH, pyrazole), 121.5 (CH, benzo[*d*][1,3]dioxole), 118.8 (2×CH, Ar), 118.6 (CH, imidazole), 108.5 (CH, benzo[*d*][1,3]dioxole), 108.3 (CH, benzo[*d*][1,3]dioxole), 101.3 (CH_2_, benzo[*d*][1,3]dioxole), 41.9 (CH_2_). [ESI‐HRMS] calculated for C_20_H_17_N_4_O_2_: 345.1352 [M+H]^+^. Found: 345.1366 [M+H]^+^.

### 3‐(4‐((1*H*‐Imidazol‐1‐yl)methyl)‐1‐phenyl‐1*H*‐pyrazol‐3‐yl)‐2‐chloro‐1*H*‐indole (10 j)

Prepared from 2‐chloro‐3‐(4‐(chloromethyl)‐1‐phenyl‐1*H*‐pyrazol‐3‐yl)‐1*H*‐indole (**9 j**) (0.80 g, 2.33 mmol). The product was eluted with CH_2_Cl_2_−MeOH 97 : 3 v/v to give the product as a brown oil, yield 0.49 g (56 %). TLC (95 : 5 CH_2_Cl_2_/MeOH), R*f*=0.72. ^1^H NMR (CDCl_3_): δ 10.75 (s, 1H, NH), 7.78 (s, 1H, imidazole), 7.63 (d, *J*=7.7 Hz, 2H, Ar), 7.48 (d, *J*=7.4 Hz, 1H, indole), 7.35 (d, *J*=7.7 Hz, 2H, Ar), 7.32 (s, 1H, pyrazole), 7.19 (d, *J*=7.5 Hz, 1H, indole), 7.16 (t, *J*=7.7 Hz, 1H, para‐Ar), 7.05 (m, 2H, indole), 6.94 (s, 1H, imidazole), 6.75 (s, 1H, imidazole), 4.99 (s, 2H, CH_2_). ^13^C NMR (CDCl_3_): δ 144.6 (C, Ar), 139.8 (C, indole), 134.9 (C, pyrazole), 129.5 (2×CH, Ar), 129.0 (CH, pyrazole), 127.6 (C, indole), 126.8 (CH, imidazole), 122.9 (C, indole), 120.8 (CH, imidazole), 119.3 (CH, imidazole), 119.1 (2×CH, indole), 119.0 (3×CH, Ar), 118.7 (C, indole), 111.0 (2×CH, indole), 104.4 (C, pyrazole), 41.9 (CH_2_). [ESI‐HRMS] calculated for C_21_H_17_ClN_5_: 374.1172 [M+H]^+^. Found: 374.1187 [M+H]^+^.

### 4‐(4‐((1*H*‐1,2,4‐Triazol‐1‐yl)methyl)‐1‐phenyl‐1*H*‐pyrazol‐3‐yl)pyridine (11 a)

Prepared from 4‐(4‐(chloromethyl)‐1‐phenyl‐1*H*‐pyrazol‐3‐yl)pyridine (**9 a**) (0.50 g, 1.82 mmol). The product was eluted with CH_2_Cl_2_−MeOH−Et_3_N 92.5 : 7.5 : 1 v/v/v to give the product as a white solid, yield 0.27 g (48 %). M.p. 98–100 °C. TLC (9 : 1 CH_2_Cl_2_/MeOH), R*f*=0.69. ^1^H NMR (DMSO‐d_6_): *δ* 8.67 (m, 3H, 2×pyridine, 1×triazole), 8.60 (s, 1H, pyrazole), 7.98 (s, 1H, triazole), 7.90 (d, *J*=7.7 Hz, 2H, Ar), 7.78 (d, *J*=5.8 Hz, 2H, pyridine), 7.55 (t, *J*=7.7 Hz, 2H, Ar), 7.38 (t, *J*=7.2 Hz, 1H, para‐Ar), 5.60 (s, 2H, CH_2_). ^13^C NMR (CDCl_3_): *δ* 152.1 (CH, triazole), 150.6 (2x CH, pyridine), 148.3 (C, Ar), 140.0 (C, Ar), 139.5 (C, Ar), 131.0 (CH, Ar), 130.2 (2x CH, Ar), 127.5 (CH, Ar), 122.3 (2×CH, Ar), 122.1 (CH, triazole), 119.2 (2x CH, Ar), 117.0 (C, Ar), 43.5 (CH_2_). [ESI‐HRMS] calculated for C_17_H_15_N_6_: 303.1358 [M+H]^+^. Found: 303.1364 [M+H]^+^.

### 3‐(4‐((1*H*‐1,2,4‐Triazol‐1‐yl)methyl)‐1‐phenyl‐1*H*‐pyrazol‐3‐yl)pyridine (11 b)

Prepared from 3‐(4‐(chloromethyl)‐1‐phenyl‐1*H*‐pyrazol‐3‐yl)pyridine (**9 b**) (0.50 g, 1.82 mmol). The product was eluted with CH_2_Cl_2_−MeOH−Et_3_N 92.5 : 7.5 : 1 v/v/v to give the product as a yellow solid, yield 0.31 g (55 %). M.p. 110–112 °C. TLC (9 : 1 CH_2_Cl_2_/MeOH), R*f*=0.68. ^1^H NMR (DMSO‐d_6_): *δ* 8.67 (m, 3H, 2×pyridine, 1×triazole), 8.60 (s, 1H, pyrazole), 7.98 (s, 1H, triazole), 7.90 (d, *J*=7.7 Hz, 2H, Ar), 7.78 (d, *J*=5.8 Hz, 2H, pyridine), 7.55 (t, *J*=7.7 Hz, 2H, Ar), 7.38 (t, *J*=7.2 Hz, 1H, para‐Ar), 5.60 (s, 2H, CH_2_). ^13^C NMR (CDCl_3_): *δ* 152.1 (CH, triazole), 150.6 (2x CH, pyridine), 148.3 (C, Ar), 140.0 (C, Ar), 139.5 (C, Ar), 131.0 (CH, Ar), 130.2 (2x CH, Ar), 127.5 (CH, Ar), 122.3 (2×CH, Ar), 122.1 (CH, triazole), 119.2 (2x CH, Ar), 117.0 (C, Ar), 43.5 (CH_2_). [ESI‐HRMS] calculated for C_17_H_15_N_6_: 303.1358 [M+H]^+^. Found: 303.1364 [M+H]^+^. ^1^H NMR (CDCl_3_): *δ* 8.96 (s, 1H, pyrazole), 8.68 (s, 1H, triazole), 8.08 (s, 1H, pyridine), 8.05 (s, 1H, triazole), 8.02 (m, 2H, pyridine), 7.75 (d, *J*=7.6 Hz, 2H, Ar), 7.51 (t, *J*=7.4 Hz, 2H, Ar), 7.43 (t, *J*=6.2 Hz, 1H, pyridine), 7.37 (t, *J*=7.4 Hz, 1H, para‐Ar), 5.48 (s, 2H, CH_2_). ^13^C NMR (CDCl_3_): *δ* 152.5 (CH, triazole), 149.4 (CH, pyridine), 148.5 (CH, pyridine), 148.4 (C, Ar), 139.4 (C, pyrazole), 135.5 (CH, pyridine), 129.6 (2×CH, Ar), 128.8 (CH, triazole), 128.5 (C, pyrazole), 127.3 (CH, para‐Ar), 123.8 (CH, pyridine), 119.2 (2×CH, Ar), 119.1 (CH, pyrazole), 115.0 (C, pyridine), 44.2 (CH_2_). [ESI‐HRMS] calculated for C_17_H_15_N_6_: 303.1353 [M+H]^+^. Found: 303.1352 [M+H]^+^.

### 3‐((1*H*‐Imidazol‐1‐yl)methyl)‐1‐(4‐methoxyphenyl)‐5‐methyl‐1*H*‐pyrazole (23)

Prepared from 3‐(chloromethyl)‐1‐(4‐methoxyphenyl)‐5‐methyl‐1H‐pyrazole (**21**) (1 g, 4.22 mmol). The product was eluted with CH_2_Cl_2_−MeOH 97.5 : 2.5 v/v to give the product as a brown oil, yield 0.65 g (58 %). TLC (9 : 1 CH_2_Cl_2_/MeOH), R*f*=0.47. ^1^H NMR (CDCl_3_): δ 7.43 (s, 1H, imidazole), 7.16 (d, *J*=8.9 Hz, 2H, Ar), 7.08 (s, 1H, imidazole), 6.96 (d, *J*=8.8 Hz, 2H, Ar), 6.82 (s, 1H, imidazole), 6.18 (s, 1H, pyrazole), 5.08 (s, 2H, CH_2_), 3.87 (s, 3H, OCH_3_), 2.34 (s, 3H, CH_3_). ^13^C NMR (CDCl_3_): δ 159.3 (C, Ar), 147.6 (C, pyrazole), 140.7 (C, pyrazole), 137.2 (CH, imidazole), 132.4 (C, Ar), 129.4 (CH, imidazole), 126.5 (2×CH, Ar), 119.3 (CH, imidazole), 114.6 (2×CH, Ar), 105.7 (CH, pyrazole), 55.6 (OCH_3_), 44.8 (CH_2_), 12.2 (CH_3_). [ESI‐HRMS] calculated for C_15_H_17_N_4_O: 269.1397 [M+H]^+^. Found: 269.1395 [M+H]^+^.

### 5‐((1*H*‐Imidazol‐1‐yl)methyl)‐1‐(4‐methoxyphenyl)‐3‐methyl‐1*H*‐pyrazole (25)

Prepared from 5‐(chloromethyl)‐1‐(4‐methoxyphenyl)‐3‐methyl‐1*H*‐pyrazole (**22**) (1 g, 4.22 mmol). The product was eluted with CH_2_Cl_2_−MeOH 97.5 : 2.5 v/v to give the product as a brown oil, yield 0.71 g (63 %). TLC (9 : 1 CH_2_Cl_2_/MeOH), R*f*=0.42. ^1^H NMR (CDCl_3_): δ 7.94 (s, 1H, imidazole), 7.34 (d, *J*=8.9 Hz, 2H, Ar), 7.19 (s, 1H, imidazole), 7.12 (s, 1H, imidazole), 7.01 (d, *J*=8.9, 2H, Ar), 6.08 (s, 1H, pyrazole), 5.20 (s, 2H, CH_2_), 3.88 (s, 3H, OCH_3_), 2.27 (s, 3H, CH_3_). ^13^C NMR (CDCl_3_): δ 159.6 (C, Ar), 147.8 (C, pyrazole), 140.9 (C, pyrazole), 137.5 (CH, imidazole), 132.6 (C, Ar), 129.7 (CH, imidazole), 126.8 (2×CH, Ar), 119.6 (CH, imidazole), 114.7 (2×CH, Ar), 106.0 (CH, pyrazole), 55.8 (OCH_3_), 45.0 (CH_2_), 12.5 (CH_3_). [ESI‐HRMS] calculated for C_15_H_17_N_4_O: 269.1397 [M+H]^+^. Found: 269.1396 [M+H]^+^.

### 1‐((1‐(4‐Methoxyphenyl)‐5‐methyl‐1*H*‐pyrazol‐3‐yl)methyl)‐1*H*‐1,2,4‐triazole (24)

Prepared from 3‐(chloromethyl)‐1‐(4‐methoxyphenyl)‐5‐methyl‐1*H*‐pyrazole (**21**) (0.5 g, 2.11 mmol). The product was eluted with CH_2_Cl_2_−MeOH 97.5 : 2.5 v/v to give the product as a brown oil, yield 0.49 g (86 %). TLC (9 : 1 CH_2_Cl_2_/MeOH), R*f*=0.45. ^1^H NMR (CDCl_3_): δ 7.95 (s, 1H, triazole), 7.84 (s, 1H, triazole), 7.24 (m, 2H, Ar), 6.97 (m, 2H, Ar), 6.24 (s, 1H, pyrazole), 5.31 (s, 2H, CH_2_), 3.86 (s, 3H, OCH_3_), 2.33 (s, 3H, CH_3_). ^13^C NMR (CDCl_3_): δ 159.4 (C, Ar), 151.8 (CH, triazole), 146.0 (C, pyrazole), 140.7 (C, pyrazole), 132.5 (C, Ar), 126.7 (CH, triazole), 126.4 (2×CH, Ar), 114.3 (2×CH, Ar), 105.8 (CH, para‐Ar), 55.6 (OCH_3_), 47.4 (CH_2_), 12.3 (CH_3_). [ESI‐HRMS] calculated for C_14_H_16_N_5_O: 270.1349 [M+H]^+^. Found: 270.1347 [M+H]^+^.

### 1‐((1‐(4‐Methoxyphenyl)‐3‐methyl‐1*H*‐pyrazol‐5‐yl)methyl)‐1*H*‐1,2,4‐triazole (26)

Prepared from 5‐(chloromethyl)‐1‐(4‐methoxyphenyl)‐3‐methyl‐1*H*‐pyrazole (**22**) (1.0 g, 4.22 mmol). The product was eluted with CH_2_Cl_2_−MeOH 97.5 : 2.5 v/v to give the product as a brown crystalline solid, yield 1.02 g (90 %). M.p. 47–48 °C. TLC (97.5 : 2.5 CH_2_Cl_2_/MeOH), R*f*=0.43. ^1^H NMR (DMSO‐d_6_): δ 8.20 (s, 1H, triazole), 7.98 (s, 1H, triazole), 7.33 (d, *J*=8.4 Hz, 2H, Ar), 6.99 (d, *J*=8.5 Hz, 2H, Ar), 6.16 (s, 1H, pyrazole), 5.48 (s, 2H, CH_2_), 3.86 (s, 3H, OCH_3_), 2.26 (s, 3H, CH_3_). ^13^C NMR (CDCl_3_): δ 159.6 (C, Ar), 152.0 (CH, triazole), 146.2 (C, pyrazole), 140.9 (C, pyrazole), 132.7 (C, Ar), 126.9 (CH, triazole), 126.7 (2×CH, Ar), 114.4 (2×CH, Ar), 106.0 (CH, para‐Ar), 55.7 (OCH_3_), 47.6 (CH_2_), 12.6 (CH_3_). Anal. Calcd for C_14_H_15_N_5_O (269.3054): C, 62.44; H, 5.61; N, 25.99. Found: C, 62.32; H, 5.39; N, 26.07.

### General Method for the Preparation of *N*‐(3‐(1*H*‐Imidazol‐1‐yl)propyl)‐1‐phenyl‐3‐(pyridin‐3/4‐yl)‐1*H*‐pyrazole‐4‐carboxamides (13) and 1‐phenyl‐3‐(pyridin‐3/4‐yl)‐*N*‐(pyridin‐4‐ylmethyl)‐1*H*‐pyrazole‐4‐carboxamides (14)

To a stirred solution of 1‐phenyl‐3‐(pyridin‐3/4‐yl)‐1*H*‐pyrazole‐4‐carboxylic acid (**12**)^14^ (1.5 mmol) in dry DMF (15 mL), was added CDI (2.2 mmol) at room temperature with continuous stirring for 2 h. The reaction was chilled in an ice‐cooled water bath and for the preparation of **13** 1‐(3‐aminopropyl)imidazole (1.55 mmol) was added, or for the preparation of **14** 4‐(aminomethyl)pyridine (1.7 mmol) was added. The mixture was refluxed at 70 °C for 48 h, then DMF was evaporated under vacuum, and the residue was extracted with EtOAc (100 mL). The organic layer was washed with water (3×50 mL), dried (MgSO_4_) and evaporated under vacuum. The product was purified by gradient column chromatography and eluted with 5 % MeOH in CH_2_Cl_2_.

### *N*‐(3‐(1*H*‐Imidazol‐1‐yl)propyl)‐1‐phenyl‐3‐(pyridin‐4‐yl)‐1*H*‐pyrazole‐4‐carboxamide (13 a)

Prepared from 1‐phenyl‐3‐(pyridin‐4‐yl)‐1*H*‐pyrazole‐4‐carboxylic acid (**12 a**)^14^ (0.43 g, 1.62 mmol) and 1‐(3‐aminopropyl)imidazole (0.2 mL, 1.67 mmol). The product was obtained as a white solid, yield 0.2 g (33 %). M.p. 104–106 °C. TLC (9 : 1 CH_2_Cl_2_/MeOH), R*f*=0.29. ^1^H NMR (DMSO‐d_6_): *δ* 9.01 (s, 1H, pyrazole), 8.64 (d, *J*=6.0 Hz, 2H, pyridine), 8.46 (t, *J*=5.4 Hz, 1H, NH), 7.93 (d, *J*=8.4 Hz, 2H, Ar), 7.87 (d, *J*=6.1 Hz, 2H, pyridine), 7.83 (s, 1H, imidazole), 7.77 (s, 1H, imidazole), 7.59 (t, *J*=7.6 Hz, 2H, Ar), 7.43 (t, *J*=7.4 Hz, 1H, para‐Ar), 7.26 (s, 1H, imidazole), 4.07 (t, *J*=6.8 Hz, 2H, CH_2_), 3.08 (q, *J*=7.3 Hz, 2H, CH_2_), 1.98 (quint, *J*=6.8 Hz, 2H, CH_2_). ^13^C NMR (CDCl_3_): *δ* 163.0 (C=O), 150.2 (2×CH, pyridine), 148.6 (C, pyridine), 140.0 (C, Ar), 139.1 (C, pyrazole), 130.2 (CH, imidazole), 130.2 (CH, para‐Ar), 129.7 (4×CH, Ar), 127.8 (CH, pyrazole), 123.2 (2×CH, imidazole), 119.4 (2×CH, pyridine), 118.2 (C, pyrazole), 45.0 (CH_2_), 37.3 (CH_2_), 31.0 (CH_2_). [ESI‐HRMS] calculated for C_21_H_21_N_6_O: 373.1777 [M+H]^+^. Found: 373.1789 [M+H]^+^.

### *N*‐(3‐(1*H*‐Imidazol‐1‐yl)propyl)‐1‐phenyl‐3‐(pyridin‐3‐yl)‐1*H*‐pyrazole‐4‐carboxamide (13 b)

Prepared from 1‐phenyl‐3‐(pyridin‐3‐yl)‐1*H*‐pyrazole‐4‐carboxylic acid (**12 b**)^14^ (0.43 g, 1.62 mmol) and 1‐(3‐aminopropyl)imidazole (0.2 mL, 1.67 mmol). The product was obtained as a white solid, yield 0.15 g (25 %). M.p. 80–82 °C. TLC (9 : 1 CH_2_Cl_2_/MeOH), R*f*=0.27. ^1^H NMR (DMSO‐d_6_): *δ* 9.00 (s, 1H, pyridine), 8.98 (s, 1H, pyrazole), 8.59 (d, *J*=3.7 Hz, 1H, pyridine), 8.33 (t, *J*=5.5 Hz, 1H, NH), 8.23 (d, *J*=7.8 Hz, 1H, pyridine), 7.92 (d, *J*=7.8 Hz, 2H, Ar), 7.66 (s, 1H, imidazole), 7.59 (t, *J*=7.6 Hz, 2H, Ar), 7.47 (m, 1H, pyridine), 7.42 (t, *J*=7.3 Hz, 1H, para‐Ar), 7.21 (s, 1H, imidazole), 6.90 (s, 1H, imidazole), 4.05 (t, *J*=6.8 Hz, 2H, CH_2_), 3.21 (q, *J*=6.2 Hz, 2H, CH_2_), 1.95 (quint, *J*=6.8 Hz, 2H, CH_2_). ^13^C NMR (DMSO‐d_6_): *δ* 163.0 (C=O), 150.0 (2×CH, pyridine), 149.6 (CH, pyridine), 149.6 (CH, pyridine), 149.3 (C, pyrazole), 148.8 (C, pyridine), 139.4 (C, Ar), 136.4 (CH, pyridine), 131.0 (CH, pyridine), 130.3 (2×CH, Ar), 128.8 (C, pyridine), 127.8 (2×CH, pyridine), 123.6 (CH, pyrazole), 122.7 (2×CH, Ar), 120.2 (C, pyrazole), 119.3 (CH, para‐Ar), 41.9 (CH_2_). Anal. Calcd for C_21_H_20_N_6_O (372.4286): C, 67.08; H, 5.47; N, 22.35. Found: C, 66.93; H, 5.57; N, 22.44.

### 1‐Phenyl‐3‐(pyridin‐4‐yl)‐*N*‐(pyridin‐4‐ylmethyl)‐1*H*‐pyrazole‐4‐carboxamide (14 a)

Prepared from 1‐phenyl‐3‐(pyridin‐4‐yl)‐1*H*‐pyrazole‐4‐carboxylic acid (**12 a**) (0.46 g, 1.73 mmol) and 4‐(aminomethyl)pyridine (0.2 mL, 1.97 mmol). The product was obtained as a white solid, yield 0.25 g (41 %). M.p. 206–208 °C. TLC (95 : 5 CH_2_Cl_2_/MeOH), R*f*=0.25. ^1^H NMR (DMSO‐d_6_): *δ* 9.09 (s, 1H, pyrazole), 8.97 (t, *J*=5.8 Hz, 1H, NH), 8.63 (d, *J*=6 Hz, 2H, pyridine), 8.54 (d, *J*=5.9 Hz, 2H, pyridine), 7.94 (d, *J*=7.8 Hz, 2H, Ar), 7.86 (d, *J*=6.0 Hz, 2H, pyridine), 7.60 (t, *J*=7.8 Hz, 2H, Ar), 7.43 (t, *J*=7.4 Hz, 1H, para‐Ar), 7.37 (d, *J*=5.8 Hz, 2H, pyridine), 4.50 (d, *J*=5.9 Hz, 2H, CH_2_). ^13^C NMR (DMSO‐d_6_): *δ* 163.0 (C=O), 150.1 (4×CH, pyridine), 149.0 (C, pyrazole), 148.7 (C, pyridine), 140.0 (C, pyridine), 139.3 (C, Ar), 131.4 (CH, pyrazole), 130.3 (2×CH, Ar), 128.0 (CH, para‐Ar), 123.1 (2×CH, pyridine), 122.7 (2×CH, pyridine), 119.4 (2×CH, Ar), 118.4 (C, pyrazole), 42.0 (CH_2_). [ESI‐HRMS] calculated for C_21_H_18_N_5_O: 356.1511 [M+H]^+^. Found: 356.1523 [M+H]^+^.

### 1‐Phenyl‐3‐(pyridin‐3‐yl)‐*N*‐(pyridin‐4‐ylmethyl)‐1*H*‐pyrazole‐4‐carboxamide (14 b)

Prepared from 1‐phenyl‐3‐(pyridin‐3‐yl)‐1*H*‐pyrazole‐4‐carboxylic acid (**12 a**) (0.51 g, 1.91 mmol) and 4‐(aminomethyl)pyridine (0.22 mL, 2.17 mmol). The product was obtained as a white solid, yield 0.32 g (48 %). M.p. 206–208 °C. TLC (9 : 1 CH_2_Cl_2_/MeOH), R*f*=0.88. ^1^H NMR (DMSO‐d_6_): *δ* 9.10 (s, 1H, pyridine), 9.00 (s, 1H, NH), 8.91 (t, *J*=5.6 Hz, 1H, pyridine), 8.58 (d, *J*=3.8 Hz, 1H, pyridine), 8.53 (d, *J*=4.9 Hz, 2H, pyridine), 8.21 (d, *J*=7.8 Hz, 1H, pyridine), 7.92 (d, *J*=7.8 Hz, 2H, Ar), 7.59 (t, *J*=7.7 Hz, 2H, Ar), 7.45 (m, 2H, pyridine), 7.36 (t, *J*=7.5 Hz, 1H, para‐Ar), 4.49 (d, *J*=5.8 Hz, 2H, CH_2_). ^13^C NMR (DMSO‐d_6_): *δ* 163.0 (C=O), 150.0 (2×CH, pyridine), 149.6 (CH, pyridine), 149.6 (CH, pyridine), 149.3 (C, pyrazole), 148.8 (C, pyridine), 139.4 (C, Ar), 136.4 (CH, pyridine), 131.0 (CH, pyridine), 130.3 (2×CH, Ar), 128.8 (C, pyridine), 127.8 (2×CH, pyridine), 123.6 (CH, pyrazole), 122.7 (2×CH, Ar), 120.2 (C, pyrazole), 119.3 (CH, para‐Ar), 41.9 (CH_2_). Anal. Calcd for C_21_H_17_N_5_O (355.3982): C, 70.97; H, 4.82; N, 19.70. Found: C, 71.05; H, 4.79; N, 19.55.

### Biochemistry

**CYP121A1 Spectral binding assays for ligand** ***K***_**D**_ **determination**: CYP121A1 protein was expressed and purified as described previously.^20^ Ligand binding assays were performed by spectrophotometric titration using a Cary 60 UV‐visible scanning spectrophotometer (Agilent, UK) and a 1 cm path length quartz cuvette, recording spectra between 250 and 800 nm. Titrations were typically done with 3–5 μM CYP121A1 at 25 °C in 100 mM potassium phosphate (KPi) buffer, 200 mM KCl, pH 7.85 with 0.004 % Triton X‐100. Ligand stock solutions were prepared in dimethylsulfoxide (DMSO). Ligands were added in small volumes (typically 0.05–0.2 μL aliquots) from concentrated stock solutions to the protein in a 1 mL final volume. Spectral measurements were taken before ligand addition, and following addition of each aliquot of ligand until no further spectral change occurred. Difference spectra at each stage in the titration were obtained by subtraction of the initial ligand‐free enzyme spectrum from subsequent spectra collected after each addition of ligand. From the difference spectra, a pair of wavelengths were identified and defined as the absorbance maximum (A_peak_) and minimum (A_trough_). The overall absorbance change (ΔA_max_) was calculated by subtracting the A_trough_ value from the A_peak_ value for each spectrum collected after a ligand addition. Graphs of ΔAmax against [ligand] were plotted for each titration. Titrations were done in triplicate and the final *K*_D_ value presented was determined as the average value across the three sets. The *K*_D_ values were determined by fitting the data using either a standard hyberbolic function (Equation 1) or the Hill equation (Equation 2) using Origin software (OriginLab, Northampton, MA).

(1) $$A{}_{\mathrm{obs}}=\left(A{}_{\max}{}^{*}L/\left(K{}_{d}+L\right)\right)$$

In Equation 1 (the standard hyperbolic function, the Michaelis‐Menten function adapted for ligand binding), A_obs_ is the observed absorbance change at ligand concentration L, A_max_ is the maximal absorbance change observed at apparent ligand saturation, and *K*_d_ is the dissociation constant for the binding of the ligand (the substrate concentration at which A_obs_=0.5×A_max_).

(2) $$A{}_{\mathrm{obs}}=\left(A{}_{\max}\times L{}^{n}\right)/\left(K{}^{n}+L{}^{n}\right)$$

In Equation 2 (the sigmoidal Hill equation), A_obs_ is the observed absorbance change at ligand concentration L, A_max_ is the absorbance change at apparent ligand saturation, *K* is the apparent dissociation constant, and n is the Hill coefficient, a value describing the apparent extent of cooperativity observed in ligand binding.

**Antimycobacterial Activity Assay**: *M. tuberculosis* H_37_Rv was grown in 7H9 liquid medium with 10 % Middlebrook OADC Growth Supplement enrichment (BBL/Becton‐Dickinson, Sparks, MD, USA). Cells were cultured at 37 °C until mid‐log phase was reached (OD_600nm_=0.4–0.6). After cells reached mid‐log phase, bacterial suspensions were prepared as described below and REMA assays were performed. The anti‐*M. tuberculosis* activities of the compounds were determined by the REMA (Resazurin Microtiter Assay) method.^21^ Stock solutions of the tested compounds (10 mg/mL) were prepared in DMSO and diluted in Middlebrook 7H9 broth supplemented with 10 % OADC. The microdilution of the compounds was performed in 96‐well plates to obtain final compound concentration ranges of 0.39–100 μg/mL. Rifampicin in the concentration range between 0.004–1 μg/mL was added as control. Bacterial suspensions were prepared and their turbidity adjusted to match the optical density of McFarland no. 1 standard. After a further dilution of 1 : 20 in Middlebrook 7H9 broth supplemented with OADC, 100 μL of the inoculum were added to each well of the 96‐well plate. Cultures were incubated for 7 days at 37 °C, and 30 μL of 0.01 % resazurin was added. Wells were read after 24 h for colour change and measured as the fluorescence (excitation/emission of 530/590 nm filters, respectively) in a microfluorimeter. The MIC was defined as the lowest concentration resulting in 90 % inhibition of *M. tuberculosis* growth. The presented results are representative from two independent experiments.

### Molecular Modeling and Docking

Docking studies were performed using the MOE^22^ program and Mtb CYP121A1 co‐crystallized with fluconazole (PDB 2IJ7). All minimisations were performed with MOE until a RMSD gradient of 0.01 Kcal/mol/A with the MMFF94 forcefield and partial charges were automatically calculated. The charge of the heme iron at physiological pH was set to 3^+^ (geometry d2sp3) through the atom manager in MOE. The Alpha Triangle placement, which derives poses by random superposition of ligand atom triplets through alpha sphere dummies in the receptor site, was chosen to determine the poses. The London ΔG scoring function estimates the free energy of binding of the ligand from a given pose. Refinement of the results was done using the MMFF94 forcefield, and rescoring of the refined results using the London ΔG scoring function was applied. The output database dock file was created with different poses for each ligand and arranged according to the final score function (S), which is the score of the last stage that was not set to zero.

### Crystallography Studies on CYP121A1

Untagged CYP121A1 protein and crystals were prepared as previously reported, with the following adaptations.^20^ Crystals were prepared using a Mosquito pipetting robot (Molecular Dimensions, Newmarket, UK) in 800 nL drops with protein‐to‐mother liquor at a ratio of 1 : 1, by vapour diffusion in 1.5–2.1 M ammonium sulfate and 0.1 M sodium MES, or Cacodylate from pH 5.5–6.15. Co‐crystals were prepared following incubation with 2 mM ligand prepared in DMSO. Protein solutions were centrifuged at 14,000 rpm for 20 mins at 4 °C immediately before crystallogenesis. Ligand soaks were also carried out either by directly dissolving solid ligand to saturation, or by the addition of a 2–5 mM ligand solution in DMSO to the mother liquor, and soaking was carried out for a minimum period of 24 h. Crystals were immersed in mother liquor supplemented with 10–30 % oil as cryoprotectant, and cryoprotected and flash‐cooled in liquid nitrogen. Data were collected on beamline i02 (wavelength 0.9795 Å) at the Diamond Light Source Facility (Harwell, UK). The diffraction data were reduced, scaled and merged using XDS or Xia2.^27^, ^28^ Structures were refined using PHENIX^29^ with the native CYP121A1 structure (PDB 1 N40)^20^ as the starting model. Structural rebuilding and validation were performed with COOT,^30^ Molprobity^31^ and PDB REDO.^32^

## Conflict of interest

The authors declare no conflict of interest.

## Supporting information

As a service to our authors and readers, this journal provides supporting information supplied by the authors. Such materials are peer reviewed and may be re‐organized for online delivery, but are not copy‐edited or typeset. Technical support issues arising from supporting information (other than missing files) should be addressed to the authors.

Supplementary
