# Supplementary material for: Design and Synthesis of Imidazole and Triazole Pyrazoles as Mycobacterium Tuberculosis CYP121A1 Inhibitors
Source: ChemistryOpen. 2019 Jul 23;8(7):995–1011. doi: 10.1002/open.201900227 (PMC6646865; doi:10.1002/open.201900227)
Supplement: Supplementary file 1 — Supplementary [file OPEN-8-995-s001.pdf]

Open Access
ChemistryOPEN

Supporting Information

© Copyright Wiley-VCH Verlag GmbH & Co. KGaA, 69451 Weinheim, 2019

Design and Synthesis of Imidazole and Triazole Pyrazoles as *Mycobacterium Tuberculosis* CYP121A1 Inhibitors

Safaa M. Kishk, Kirsty J. McLean, Sakshi Sood, Darren Smith, Jack W.D. Evans, Mohamed A. Helal, Mohamed S. Gomaa, Ismail Salama, Samia M. Mostafa, Luiz Pedro S. de Carvalho, Colin W. Levy, Andrew W. Munro, and Claire Simons*©201x The Authors. Published by Wiley-VCH Verlag GmbH & Co. KGaA.
This is an open access article under the terms of the Creative Commons Attribution License, which permits use, distribution and reproduction in any medium, provided the original work is properly cited.

Figure S1. Electron density maps of **10j** S2

Figure S2. Electron density map of **14a** S2

Figure S3. Representative images of selected Series 1 extended pyridyl compounds S3

Figure S4. Representative images of selected Series 2 compounds S4

Figure S5. Overlapped images from molecular modelling S4

Table S1. X-ray crystallography data collection and final structural refinement statistics for CYP121A1 in complex with compounds **10j** and **14a**. S5

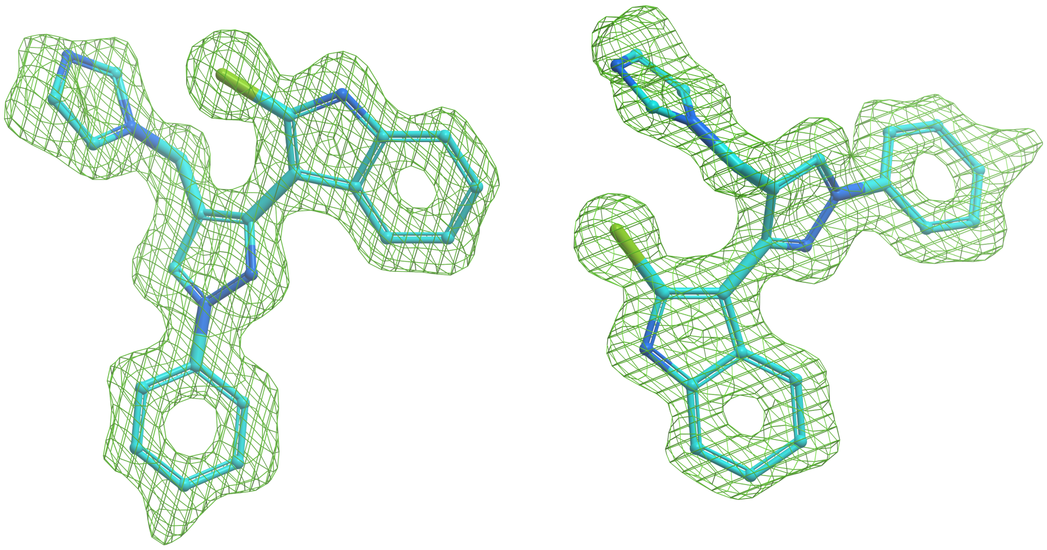

Figure S1. Electron density maps of **10j** (PDB 6GEO) (fo-fc omit electron density contoured at 2 sigma.)

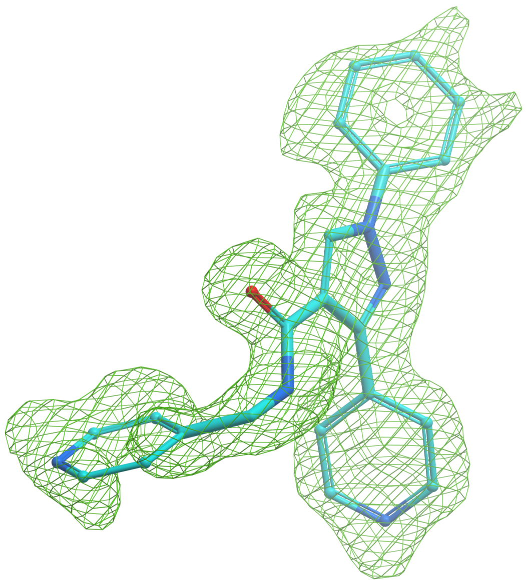

Figure S2. Electron density map of **14a** (PDB 6GEQ) (fo-fc omit electron density contoured at 2 sigma.)

A

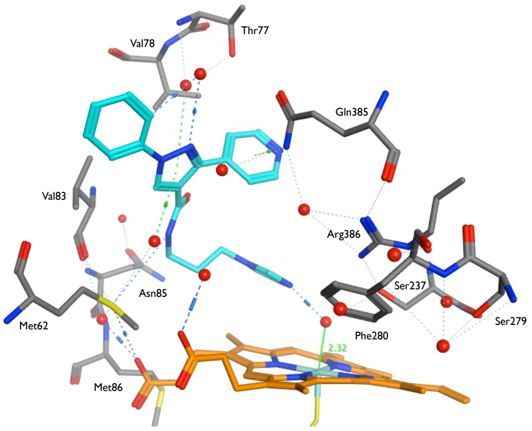

B

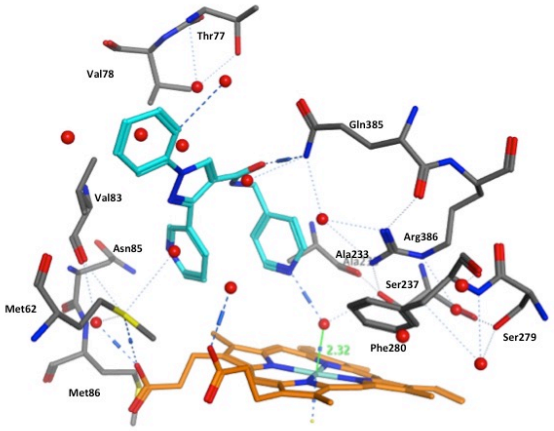

Figure S3. Representative images of selected Series 1 extended pyridyl compounds. (A) The imidazole ring of **13a** interacts with the heme iron through an interstitial water molecule bonded with Ser237. The 4-pyridine ring forms a H-bond with Gln385 and direct and indirect bonds are observed with the pyrazole ring. (B) The 4-pyridyl ring of **14b** interacts with Ser237 via an interstitial water molecule and through an arene-H interaction with Ala233. A H-bond with Gln385 is also formed with the carbonyl group of **14b**.

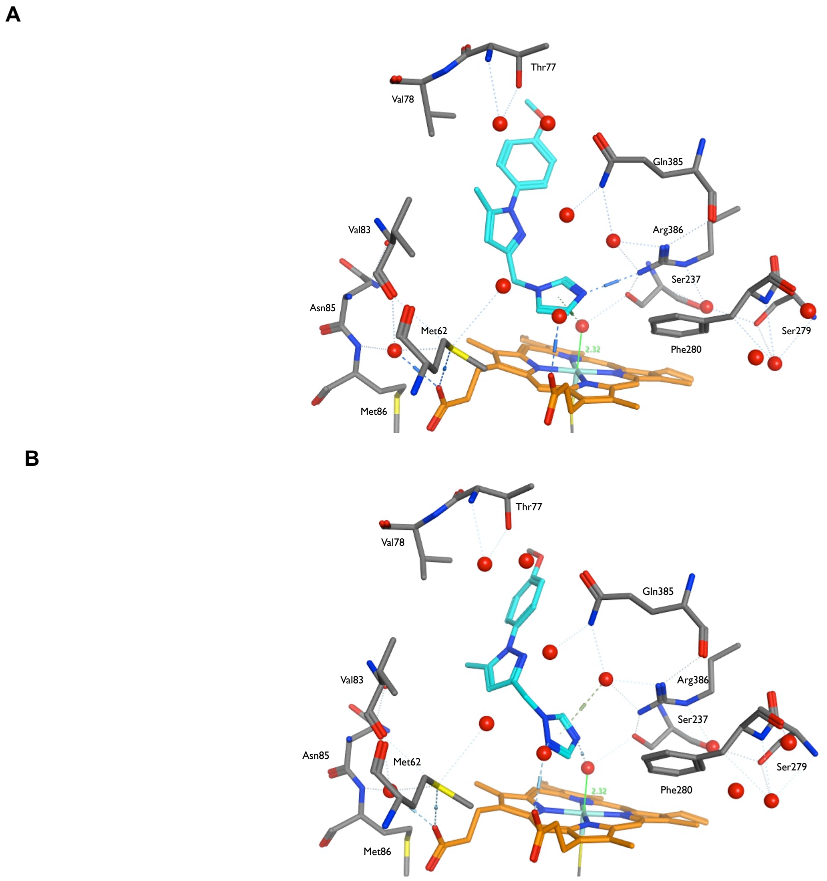

Figure S4. Representative images of selected Series 2 compounds. (A) The imidazole group of **23** interacts with the heme iron indirectly via an interstitial water molecule and directly with Arg386 via a hydrogen bond interaction (B) The triazole derivative **26** interacts with the heme iron indirectly via an interstitial water molecule, and also hydrogen bonds to Ser237 and other residues through an interstitial water.

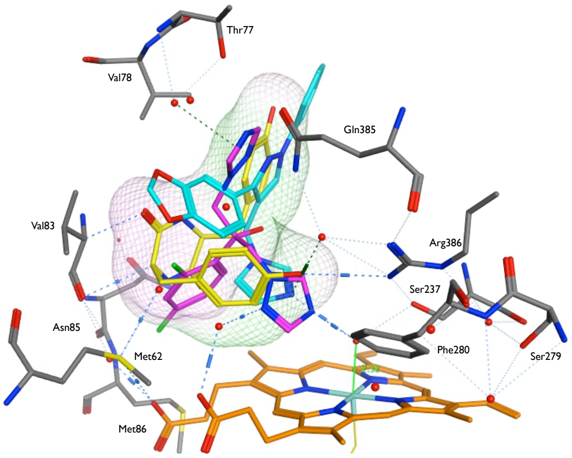

Figure S5. Overlapped images from molecular modelling of benzo[*d*][1,3]dioxole imidazole (**10i**, cyan), cYY (yellow) and fluconazole (magenta) within the CYP121A1 active site.

Table S1. X-ray crystallography data collection and final structural refinement statistics for CYP121A1 in complex with compounds **10j** and **14a**.

Data Collection and Refinement[a]	CYP121A1/**10j** complex PDB ID 6GEO	CYP121A1/**14a** complex PDB ID 6GEQ
Wavelength (Å)	0.9795	0.9795
Resolution range (Å)	44.01 - 1.5 (1.554 - 1.5)	53.35 - 1.6 (1.657 - 1.6)
Space group	P 65 2 2	P 65 2 2
Unit cell		
a, b, c (Å)	77.8073 77.8073 264.053	77.4466 77.4466 264.034
α, β, γ (°)	90 90 120	90 90 120
Total reflections	1382551 (103113)	1144991 (114021)
Unique reflections	76715 (7491)	62925 (6138)
Multiplicity	18.0 (13.8)	18.2 (18.6)
Completeness (%)	98.59 (99.59)	99.37 (99.63)
Mean I/sigma(I)	22.00 (5.45)	11.03 (2.64)
Wilson B-factor (Å^2)	12.86	15.6
R-merge	0.07361 (0.3171)	0.1593 (0.5184)
R-meas	0.07574 (0.3294)	0.1639 (0.533)
R-pim	0.01764 (0.08773)	0.03821 (0.1231)
CC1/2	0.999 (0.972)	0.998 (0.946)
CC*	1 (0.993)	0.999 (0.986)
Reflections used in refinement	75639 (7460)	62533 (6115)
Reflections used for R-free	3837 (395)	3177 (295)
R-work	0.1671 (0.2081)	0.1715 (0.2216)
R-free	0.1823 (0.2354)	0.2051 (0.2641)
CC(work)	0.964 (0.943)	0.959 (0.899)
CC(free)	0.961 (0.907)	0.952 (0.851)
No. of non-hydrogen atoms	3787	3857
macromolecules	3058	3187
ligands	85	80
solvent	644	590
Protein residues	395	396
RMS(bonds)	0.009	0.007
RMS(angles)	1.29	1.22
Ramachandran favored (%)	98.98	99.49
Ramachandran allowed (%)	1.02	0.51
Ramachandran outliers (%)	0	0
Rotamer outliers (%)	1.54	1.75
Clashscore	3.5	3.35
Average B-factor	16.29	19.19
macromolecules	13.98	17.16
ligands	15.01	16.97
solvent	27.4	30.46

[a]Data for the highest resolution shell are shown in parentheses
